# Crystal structures of two Sm^III^ complexes with dipicolinate [DPA]^2−^ ligands: comparison of luminescent properties of products obtained at different pH values

**DOI:** 10.1107/S2056989023004814

**Published:** 2023-06-09

**Authors:** Sabina Svava Mortensen, Thomas Just Sørensen

**Affiliations:** a Department of Chemistry & Nano-Science Center, University of Copenhagen, Universitetsparken 5, 2100 København Ø, Denmark; Vienna University of Technology, Austria

**Keywords:** crystal structure, luminescence, samarium(III), PXRD, hydrogen bonding

## Abstract

The formation of the two title compounds, Na_3_[Sm(DPA)_3_]·14H_2_O tris­odium tris­(pyridine-2,6-di­carboxyl­ato-κ^3^ *O* ^2^,*N*,*O* ^6^)samarate(III) tetra­deca­hydrate, Na_3_[Sm(C_7_H_3_NO_4_)_3_]·14H_2_O, and *catena*-poly[[[di­aqua­(6-carb­oxy­pyridine-2-carb­oxyl­ato-κ^3^ *O* ^2^,*N*,*O* ^6^)samarium(III)]-μ-pyridine-2,6-di­carboxyl­ato-κ^4^ *O* ^2^,*N*,*O* ^6^:*O* ^2^] tetra­hydrate], {[Sm(C_7_H_3_NO_4_)(C_7_H_4_NO_4_)(H_2_O)_2_]·4H_2_O}_*n*_, depends on the pH value adjusted with NaOH solution. In both crystal structures, the coordination spheres of the Sm^III^ cations were found to be best described by a tricapped trigonal prism (TTP), with a more regular O_6_N_3_ donor set for Na_3_[Sm(DPA)_3_]·14H_2_O than that of O_7_N_2_ for [Sm(DPA)(HDPA)(H_2_O)_2_]·4H_2_O. The supra­molecular features of both crystal structures are dominated by O—H⋯O hydrogen bonds between water mol­ecules and the O atoms of the dipicolinato ligands. Samples were made from solutions at pH = 2, pH = 5, pH = 7, and pH = 10, and the crystals present in each sample were ground to a powder. The powder samples were analyzed with powder X-ray diffraction and luminescence spectroscopy. The splitting of the bands in the luminescence spectra recorded on powders at 77 K was observed to vary with the pH.

## Abstract 2

Crystallization of the title compounds, synthesized from Sm(CF_3_SO_3_)_3_ and dipicolinic acid solution, was found to change as a function of pH.

## 1. Chemical context

The luminescent properties of lanthanide(III) complexes involving the tridentate dipicolinato ligand, [DPA]^2–^, have been studied in great detail (Aebischer *et al.*, 2009 ▸; Brayshaw *et al.*, 1995 ▸; Chauvin *et al.*, 2004 ▸; Kim *et al.*, 1998 ▸; Kofod *et al.*, 2020 ▸; Mondry & Starynowicz, 1995 ▸; Murray *et al.*, 1990 ▸; Salaam *et al.*, 2022 ▸; Zhou *et al.*, 1994 ▸). The luminescent characteristics can be explained by the fact that the formed lanthanide(III) complex with three [DPA]^2–^ ligands coordinating to the central lanthanide(III) cation exhibits an almost perfect tricapped trigonal prism (TTP) coordination environment (Albertsson, 1970 ▸; Brayshaw *et al.*, 1995 ▸; Kim *et al.*, 1998 ▸; Li *et al.*, 2019 ▸; Murray *et al.*, 1990 ▸; Salaam *et al.*, 2022 ▸; Zhou *et al.*, 1994 ▸). The luminescence properties have been studied in great depth; however, our knowledge of Sm^III^ with [DPA]^2–^ as the ligand is rather limited (Chuasaard *et al.*, 2017 ▸; Kumar *et al.*, 2019 ▸; Sharif *et al.*, 2016 ▸; Viveros-Andrade *et al.*, 2017 ▸).

In the present communication, we report the crystal structures of two compounds with Sm^III^ cations and [DPA]^2–^ ligands, *viz*. salt-like Na_3_[Sm(DPA)_3_]·14H_2_O and polymeric [Sm(DPA)(HDPA)(H_2_O)_2_]·4H_2_O. Both crystallized from mixtures of Sm(CF_3_SO_3_)_3_ and H_2_DPA solutions at different pH values adjusted with NaOH solution. The amount of the two compounds crystallized in each sample was found to be controlled by the pH value. This behavior was also monitored by powder X-ray diffraction (PXRD) of the bulk products and their luminescence spectra. 
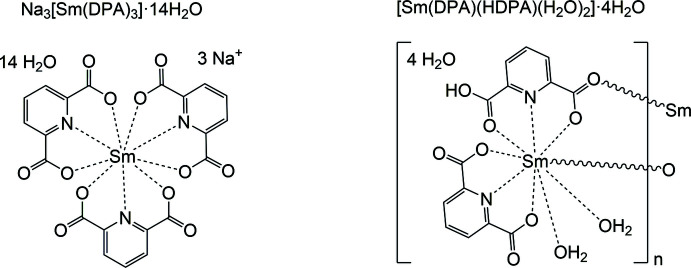


## 2. Structural commentary

**Figure 1:**
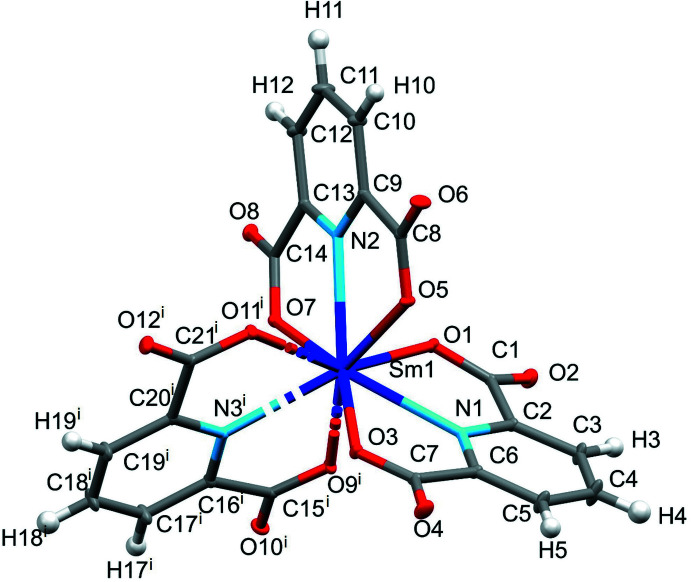
Coordination around the Sm^III^ cation in Na_3_[Sm(DPA)_3_]·14H_2_O. Displacement ellipsoids are drawn at the 50% probability level. [Symmetry code: (i) *x*, *y* + 1, *z*.]

Fig. 1 ▸ shows the coordination environment of the central Sm^III^ cation in the crystal structure of Na_3_[Sm(DPA)_3_]·14H_2_O (CCDC number: 2246128). The donor set consists of six oxygen atoms from three chelating [DPA^2–^] ligands, forming the top and bottom plane of a trigonal prism, and of three capping nitro­gen donor atoms placed in the central plane of the trigonal prism. Each of the four Na^I^ cations (two on general positions and two on inversion centers) coordinates by aqua ligands and carboxyl­ate O atoms, thus linking the [Sm(DPA)_3_]^3–^ anions into a tri-periodic structure.

**Figure 2:**
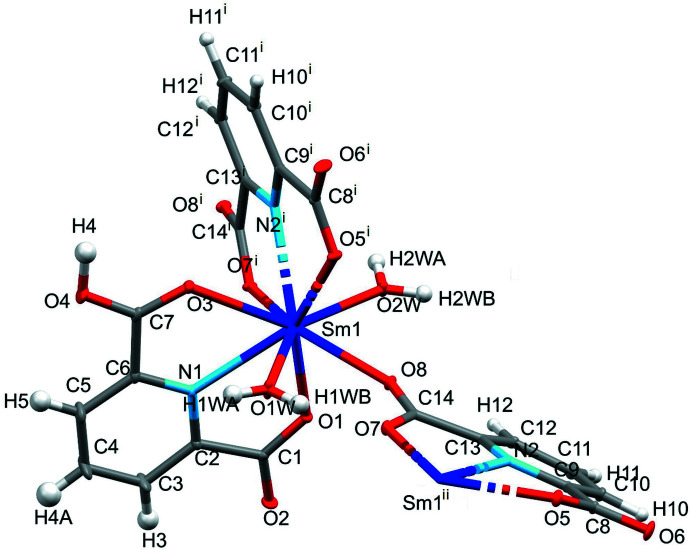
Coordination around the Sm^III^ cation in [Sm(DPA)(HDPA)(H_2_O)_2_]·4H_2_O. Displacement ellipsoids are drawn at the 50% probability level. [Symmetry codes: (i) *x*, −*y* + 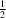 , *z* + 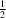 ; (ii) *x*, −*y* + 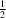 , *z* − 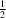 .]

Fig. 2 ▸ illustrates the coordination environment of the central Sm^III^ cation in the crystal structure of [Sm(DPA)(HDPA)(H_2_O)_2_]·4H_2_O(CCDC number: 2246127). Although the coordination number of 9 is the same as in Na_3_[Sm(DPA)_3_]·14H_2_O, here the donor set consists of seven oxygen atoms and two nitro­gen atoms. Both [HDPA]^−^ and [DPA]^2–^ ligands *N*,*O*,*O*′-chelate the metal cation. The coordination sphere is completed by two aqua ligands and the carboxyl­ate O atom of another symmetry-related [DPA]^2–^ anion, making it a polymeric structure, with chains of mol­ecules extending parallel to [001].

For both coordination environments of the Sm^III^ cation in the title compounds, a symmetry-deviation analysis was performed to determine the deviation from an ideal coordin­ation environment for coordination number 9. This was achieved by calculating a symmetry-deviation value, *σ*_ideal_, using *AlignIt* (Storm Thomsen *et al.*, 2022 ▸). More details of this method are described in the supporting information. Na_3_[Sm(DPA)_3_]·14H_2_O was found to be best described as having the shape of a tricapped trigonal prism (TTP), with *σ*_ideal_ = 1.16, which is in good agreement with what has been reported for other isostructural *Ln* ^III^ complexes (Albertsson, 1970 ▸, 1972 ▸; Hojnik *et al.*, 2015 ▸; Mondry & Starynowicz, 1995 ▸; Tancrez *et al.*, 2005 ▸; Elahi & Rajasekharan, 2016 ▸). The donor set in [Sm(DPA)(HDPA)(H_2_O)_2_]·4H_2_O is less symmetric compared to Na_3_[Sm(DPA)_3_]·14H_2_O, consisting of two nitro­gen atoms and seven oxygen atoms. Nevertheless, the Sm^III^ cation in [Sm(DPA)(HDPA)(H_2_O)_2_]·4H_2_O was also found to have a coordination polyhedron derived from a TTP, with *σ*_ideal_ = 0.73. This is in good agreement with what was reported for the Eu^III^ analogue (Liu *et al.*, 2014 ▸).

## 3. Supra­molecular features

**Figure 3:**
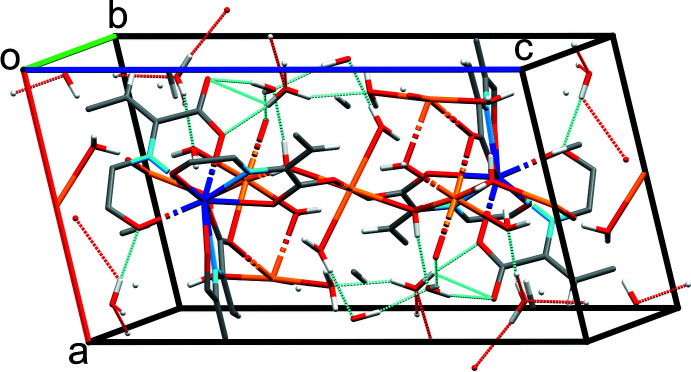
Hydrogen-bonding network in the Na_3_[Sm(DPA)_3_]·14H_2_O unit cell. Color code: Sm = dark blue, N = light blue, C = gray, H = white, O = red and Na = orange.

Both Na_3_[Sm(DPA)_3_]·14H_2_O and [Sm(DPA)(HDPA)(H_2_O)_2_]·4H_2_O contain water mol­ecules, either solely present as solvent mol­ecules for the Na-containing phase (14 per formula unit), or as solvent mol­ecules and as ligands (2 and 4, respectively) for the other phase. Hence, the packing of the structural entities is mainly consolidated by O—H⋯O hydrogen-bonding networks (Tables 1 ▸, 2 ▸; Figs. 3 ▸, 4 ▸). The shortest hydrogen bonds in the two structures are formed between the carboxyl­ate and carb­oxy­lic acid groups in [DPA]^2–^ and [HDPA]^−^ to the water mol­ecules, including, for example, O4*W*—H4*WB*⋯O5*W*, O8*W*—H8*WA*⋯O10^vii^, and O9*W*—H9*WB*⋯O1^viii^ in the Na_3_[Sm(DPA)_3_]·14H_2_O structure, and O1*W*—H1*WB*⋯O7, O4*W*—H4*WA*⋯O2^v^, and O6*W*—H6*WB*⋯O6 in the [Sm(DPA)(HDPA)(H_2_O)_2_]·4H_2_O structure. Notably, in [Sm(DPA)(HDPA)(H_2_O)_2_]·4H_2_O a very strong hydrogen bond [O4⋯O4*W* = 2.4703 (19) Å] is established between the carb­oxy group of the [HDPA]^−^ ligand and a solvent water mol­ecule.

## 4. Phase formation at different pH values

**Figure 5:**
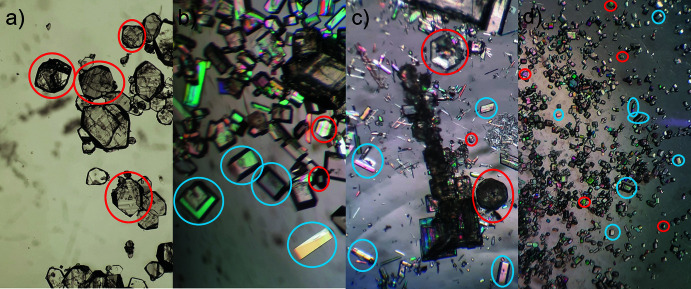
Crystal photographs from selected samples obtained at different pH values; [Sm(DPA)(HDPA)(H_2_O)_2_]·4H_2_O crystals are circled in red and Na_3_[Sm(DPA)_3_]·14H_2_O in blue. (*a*) Crystals obtained from a solution at pH = 2, where [Sm(DPA)(HDPA)(H_2_O)_2_]·4H_2_O dominates. (*b*) Crystals obtained from a solution at pH = 5, where an almost equal distribution of [Sm(DPA)(HDPA)(H_2_O)_2_]·4H_2_O and Na_3_[Sm(DPA)_3_]·14H_2_O was found. (*c*) Crystals obtained from a solution at pH = 7, where more Na_3_[Sm(DPA)_3_]·14H_2_O than [Sm(DPA)(HDPA)(H_2_O)_2_]·4H_2_O crystallized. (*d*) Crystals obtained from a solution at pH = 10, where Na_3_[Sm(DPA)_3_]·14H_2_O dominates. The images in each panel were selected from three crystallization batches performed at each pH value.

**Figure 6:**
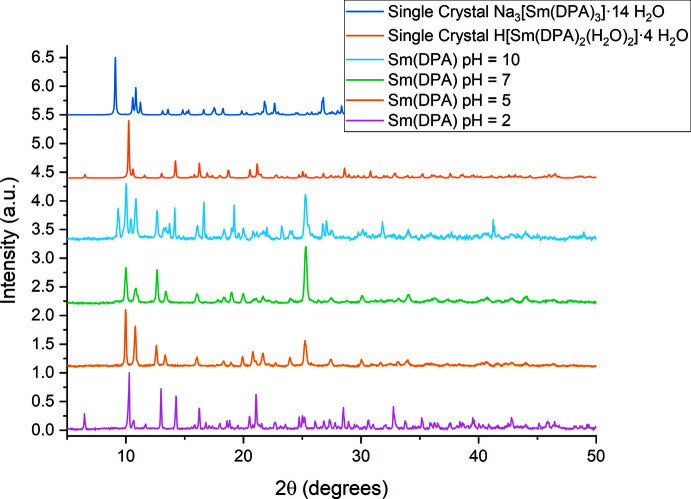
PXRD pattern of the bulk for samples prepared from solution at pH = 2, pH = 5, pH = 7, and pH = 10, as well as simulated PXRD pattern on basis of the current single-crystal data.

By combining a solution of Sm(CF_3_SO_3_)_3_ and H_2_DPA solutions at different pH values, the two title compounds crystallized in each batch. However, the amounts of each compound in a batch were found to be dependent on the pH value of the H_2_DPA solution, which was controlled by addition of NaOH solution. Samples were made at pH = 2, pH = 5, pH = 7, and pH = 10, and the varying amount of the two compounds could be observed from the crystal photographs of each batch (Fig. 5 ▸). [Sm(DPA)(HDPA)(H_2_O)_2_]·4H_2_O was the dominating compound at pH = 2 (Fig. 5 ▸ *a*), while Na_3_[Sm(DPA)_3_]·14H_2_O was found to dominate at pH = 5, pH = 7, and pH = 10 (Fig. 5 ▸ *b*,*c*,*d*). This finding is supported by PXRD data recorded from crystals crushed to a powder for all samples (Fig. 6 ▸).

## 5. Analysis of luminescence spectra for samples obtained at different pH values

The crystal field splitting is sensitive to the coordination environment and the donor atoms (Eliseeva & Bünzli, 2010 ▸). As Sm^III^ is a luminescent lanthanide(III) cation, the crystal field splitting of the spin-orbit defined *^S^L_J_* term into the individual electronic states, here double-degenerate Kramers doublets defined by ±*m_J_* values, can be observed from the luminescence spectra (Cheisson & Schelter, 2019 ▸; Wybourne, 2004 ▸; Chen *et al.*, 2005 ▸; Mortensen *et al.*, 2022 ▸; Carnall *et al.*, 1968 ▸). Because Sm^III^ is a Kramers cation, it has an uneven number of electrons (4*f* ^5^) and all states will be double degenerate without the presence of a magnetic field (Eliseeva & Bünzli, 2010 ▸). The electronic states in Sm^III^ are a complicated ^6^H_5/2_ ground state and a ^4^G_5/2_ emitting state that both have a large multiplicity (Eliseeva & Bünzli, 2010 ▸; Chen *et al.*, 2005 ▸). The emitting state, ^4^G_5/2_, can split into maximum Kramers levels. The maximum splitting is calculated as (2*J* + 1)/2 (Eliseeva & Bünzli, 2010 ▸). For the states observed from the emission spectra, the maximum splitting is three, four, five, and six for ^6^H_5/2_, ^6^H_7/2_, ^6^H_9/2_, and ^6^H_11/2_, respectively. To avoid deconvolution of nine bands or more in each trans­ition, the spectra were recorded at 77 K for the polycrystalline material. At 77 K, one of the ±*m_J_* doublets is predominately populated in ^4^G_5/2_. Thus, only three bands will be observed for the ^4^G_5/2_ → ^6^H_5/2_ transition (Lupei *et al.*, 2012 ▸; Chen *et al.*, 2005 ▸; Skaudzius *et al.*, 2018 ▸; Sakirzanovas *et al.*, 2011 ▸). The number of observed bands for a ^4^G_5/2_ → ^6^H_*J*_ transition should correspond to the maximum splitting of ^6^H_*J*_ (Eliseeva & Bünzli, 2010 ▸; Lupei *et al.*, 2012 ▸; Chen *et al.*, 2005 ▸; Skaudzius *et al.*, 2018 ▸; Sakirzanovas *et al.*, 2011 ▸). Additional bands can be an indicator for transitions from the less populated higher-energy ^4^G_5/2_ states or the presence of more than one emitting species (Chen *et al.*, 2005 ▸; Sakirzanovas *et al.*, 2011 ▸). Because both Na_3_[Sm(DPA)_3_]·14H_2_O and [Sm(DPA)(HDPA)(H_2_O)_2_]·4H_2_O are present in the samples, more bands than the maximum splitting are expected (Judd, 1962 ▸; Ofelt, 1962 ▸).

**Figure 7:**
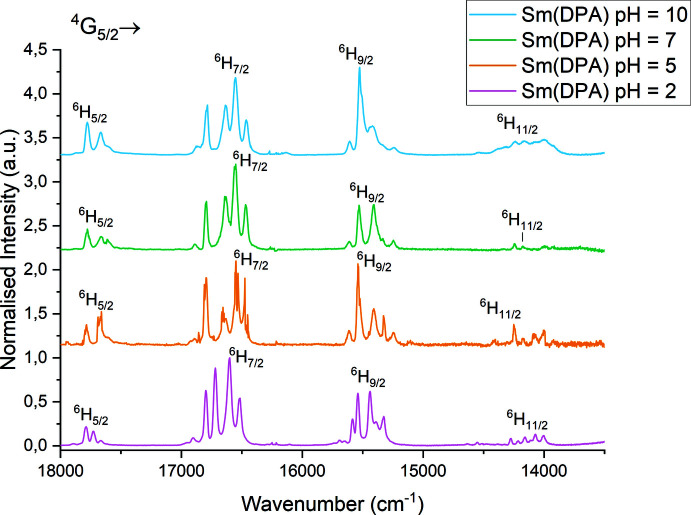
Normalized emission spectra in 2-methyltetra­hydro­furan glass at 77 K (excitation at 394 nm) for samples prepared at pH = 2, pH = 5, pH = 7, and pH = 10.

The luminescent properties of the samples obtained at different pH values were investigated in order to evaluate the effect of having different compounds present in each sample. From the emission spectra it was apparent that there is a change in the luminescenct properties with the change in compound distribution at different pH values (Fig. 7 ▸).

Five bands are observed for the ^4^G_5/2_ → ^6^H_7/2_ transition in the emission spectra from all samples. This indicates that more than one species is present in the solid reaction product, as this is one more band than the maximum splitting for ^6^H_7/2_ would allow per Sm^III^ atom. Hence, Sm^III^ exists in more than one coordination environment in the powdered samples of the bulk material. Thermal populations of more Kramers levels in ^4^G_5/2_ would result in eight bands (Lupei *et al.*, 2012 ▸; Chen *et al.*, 2005 ▸; Skaudzius *et al.*, 2018 ▸, Sakirzanovas *et al.*, 2011 ▸). As this is not the case, the five bands are ascribed as a result of the presence of both Na_3_[Sm(DPA)_3_]·14H_2_O and [Sm(DPA)(HDPA)(H_2_O)_2_]·4H_2_O, which both have a significant contribution to the emission spectrum of the samples obtained at different pH.

The change in the luminescent properties is apparent in the ^4^G_5/2_ → ^6^H_9/2_ transition, where the splitting patterns clearly varies. Additionally, there is a change in the intensity of the ^4^G_5/2_ → ^6^H_9/2_ transition compared to the the ^4^G_5/2_ → ^6^H_7/2_ transition. At pH = 2, the ^4^G_5/2_ → ^6^H_7/2_ transition is the most intense, whereas at pH = 10, the ^4^G_5/2_ → ^6^H_9/2_ transition has a higher intensity compared to ^4^G_5/2_ → ^6^H_7/2_ transition. Also, the ^4^G_5/2_ → ^6^H_5/2_ transition increased in intensity compared to the ^4^G_5/2_ → ^6^H_7/2_ band with increasing pH. However, no clear spectral components could be assigned to either Na_3_[Sm(DPA)_3_]·14H_2_O or [Sm(DPA)(HDPA)(H_2_O)_2_]·4H_2_O. Additional spectra are included in the supporting information.

## 6. Database survey

Na_3_[Sm(DPA)_3_]·14H_2_O is isostructural with other Na_3_[*Ln*(DPA)_3_]·14H_2_O compounds previously reported for La^III^, Ce^III^, Pr^III^, Nd^III^, Sm^III^, Eu^III^, Gd^III^, Tb^III^, Dy^III^, Ho^III^, Yb^III^, and Lu^III^ (Albertsson, 1970 ▸; Hojnik *et al.*, 2015 ▸; Albertsson, 1972 ▸; Albertsson *et al.*, 1972 ▸; Mondry & Starynowicz, 1995 ▸; Tancrez *et al.*, 2005 ▸; Elahi & Rajasekharan, 2016 ▸). Crystal data of Na_3_[Sm(DPA)_3_]·14H_2_O have been deposited at the CCDC (CSD code SOPGOT; Hu *et al.*, 1989 ▸); however, without atomic coordinates, which motivated us to reinvestigate the crystal structure.

[Sm(DPA)(HDPA)(H_2_O)_2_]·4H_2_O is isostructural with other [*Ln*(DPA)(HDPA)(H_2_O)_2_]·4H_2_O compounds prev­iously reported for Ce^III^, Pr^III^, Nd^III^, Sm^III^, Eu^III^, Gd^III^, Tb^III^, Dy^III^, and Er^III^ (Brayshaw *et al.*, 2005 ▸; Cheng *et al.*, 2007 ▸; Chuasaard *et al.*, 2017 ▸; Ghosh & Bharadwaj, 2003 ▸; Hou *et al.*, 2011 ▸; Kang, 2011 ▸; Liu *et al.*, 2005 ▸; Moghzi *et al.*, 2020 ▸; Najafi *et al.*, 2017 ▸; Rafizadeh *et al.*, 2005 ▸; Song *et al.*, 2005 ▸; Wang *et al.*, 2012 ▸; Xu *et al.*, 2009 ▸; Kong *et al.*, 2022 ▸). The crystal structure of [Sm(DPA)(HDPA)(H_2_O)_2_]·4H_2_O has been reported prev­iously several times (CSD code FONCUH; best result in terms of reliability factors: FONCUH01; Rafizadeh *et al.*, 2005 ▸). For inter­pretation of the luminescence spectra and a comparison with Na_3_[Sm(DPA)_3_]·14H_2_O, we have also reinvestigated the crystal structure of [Sm(DPA)(HDPA)(H_2_O)_2_]·4H_2_O.

## 7. Synthesis and crystallization

All chemicals were used as received without further purification. All crystallization experiments were conducted three times.

**0.2** ***M*** **Sm(CF_3_SO_3_)_3_ stock solution**

Sm(CF_3_SO_3_)_3_ (2.39 g, 0.400 mmol; 98% from STREM Chemicals) was used to create a 0.20 *M* stock solution by dissolving the salt in water to create a solution with a volume of 20.0±0.04 ml.

**0.2** ***M*** **H_2_DPA stock solution**

H_2_DPA (pyridine-2,6-di­carb­oxy­lic acid; 0.669 g, 4.01 mmol; Riedel-De Haën) was used to create a 0.2 *M* stock solution by dissolving the acid in water to create a solution with a volume of 20±0.04 ml.

**Sm(DPA) at pH = 2 – crystallization**

1.0 ml of the 0.2 *M* Sm(CF_3_SO_3_)_3_ stock solution was added to a sample vial with 3.0 ml of the 0.2 *M* H_2_DPA stock solution. The sample was heated at 353 K for 1 h. The sample vial was closed with a lid and left in a dark place. After 1 d crystals had formed.

**Sm(DPA) at pH = 5 – crystallization**

NaOH (1.0 *M*) was added to the H_2_DPA stock solution to adjust the pH to 5. 0.5 ml of the 0.2 *M* Sm(CF_3_SO_3_)_3_ solution were added to a sample vial with 1.5 ml of the 0.2 *M* H_2_DPA stock solution. The sample was heated at 353 K for 1 h. The sample vial was placed in a container with acetone, placing a lid on top of the container and left for acetone diffusion. After 3 d crystals had formed.

**Sm(DPA) at pH = 7 – crystallization**

NaOH (1.0 *M*) was added to the H_2_DPA stock solution to adjust the pH to 7. 0.5 ml of the 0.2 *M* Sm(CF_3_SO_3_)_3_ solution were added to a sample vial with 1.5 ml of the 0.2 *M* H_2_DPA stock solution. The sample was heated at 353 K for 1 h. The sample was then filtered through a Q-Max RR syringe filter from Frisinette and transferred to a vial. The latter was placed in a container with acetone, placing a lid on top of the container and left for an acetone diffusion. After 1 d crystals had formed.

**Sm(DPA) at pH = 10 – crystallization**

NaOH (1.0 *M*) was added to the H_2_DPA stock solution to adjust the pH to 10. 0.5 ml of the 0.2 *M* Sm(CF_3_SO_3_)_3_ solution were added to a sample vial with 1.5 ml of the 0.2 *M* H_2_DPA stock solution. The sample was heated at 353 K for 1 h. The sample was then filtered through a Q-Max RR syringe filter from Frisinette and transferred to a vial. The later was placed in a container with acetone, placing a lid on top of the container and left for an acetone diffusion. After 1 d crystals had formed.

## 8. Other experimental procedures

For both PXRD and optical spectroscopy measurements, the crystals, which had precipitated in each sample, were collected by suction filtration with a vacuum pump. The crystals were removed from the filter, dried in air and ground to a powder.

**Powder X-ray Diffraction**

PXRD diffractograms were recorded for all samples prepared at different pH values. Data were collected using a Bruker D8 Advance diffractometer using a Cu *K*α source (λ = 1.5406 Å). Samples were measured using a low-background silica sample holder at 293 K.

**Optical Spectroscopy**

Crystal powders from all samples prepared at different pH were added to a 5.0 mm diameter NMR silica cylinder (Bruker) together with 2-methyl­tetra­hydro­furan glass. The samples were cooled using liquid nitro­gen and were measured using a cold-finger setup. This setup was used for both the emission and excitation spectra and for determination of luminescent lifetimes.

Emission and excitation spectra were measured with a xenon arc lamp as the excitation source on a PTI QuantaMaster8075 from Horiba Scientific.

For emission spectroscopy, an excitation wavelength at 401 nm (24938 cm^−1^) was used. Emission was detected from 550 nm (18182 cm^−1^) to 760 nm (13158 cm^−1^). The emission slits were kept at 1.0 nm for the two outermost slits and 5.0 nm or the middle slit for all samples, and the excitation slits were all kept at 8.0 nm. The voltage bias was kept at 3.2 V for the reference detector.

For excitation spectroscopy, an emission wavelength at 598 nm (16722 cm^−1^) was used. Excitation was detected from 250 nm (40000 cm^−1^) to 590 nm (16949 cm^−1^). Emission slits were all kept at 8.0 nm and excitation slits were kept at 1.0 nm for the two outermost slits and 5.0 nm for the middle slit for all samples. The voltage bias was kept at 6.8 V for the reference detector.

**Luminescence Lifetimes**

The luminescence lifetimes were determined for all powder samples using a TCSPC FluoTime300 from PicoQuant. The excitation wavelength was 405 nm (24691 cm^−1^), and the emission wavelength 600 nm (16667 cm^−1^). The effective sync rate was kept at 1 kHz, with 5000 pulses, a period length of 1.0 ms, a burst length of 625 µs, and a time/channel at 80 ns. The temperature was kept at 298 K. The luminescence lifetimes were fitted using a mono-exponential decay function using the software *EasyTau 2* (PicoQuant, 2018 ▸).

## 9. Refinement

Crystal data, data collection and structure refinement details are summarized in Table 3 ▸. Hydrogen atoms attached to aromatic carbon atoms were added automatically using a riding model with *U*_iso_(H) = 1.2*U*_eq_(C). All hydrogen atoms of water mol­ecules were discernible in difference-Fourier maps. They were refined with a distance restraint of 0.85 Å, and with *U*_iso_(H) = 1.5*U*_eq_(C). The H atom of the carboxyl­ate group (H4) in [Sm(DPA)(HDPA)(H_2_O)_2_]·4H_2_O was found in difference-Fourier maps and was refined freely. The comparatively high residual positive electron density in Na_3_[Sm(DPA)_3_]·14H_2_O is located at distances of ≃1.4 Å from atoms H6*WA* and H6*WB*. Contributions of additional atoms and/or disorder did not result in other reasonable models.

## Supplementary Material

Crystal structure: contains datablock(s) mo_d8v5334_0m_a, I, II. DOI: 10.1107/S2056989023004814/wm5678sup1.cif

Structure factors: contains datablock(s) I. DOI: 10.1107/S2056989023004814/wm5678Isup2.hkl

Structure factors: contains datablock(s) II. DOI: 10.1107/S2056989023004814/wm5678IIsup3.hkl

Spectra, symmetry deviation analysis, and PXRD. DOI: 10.1107/S2056989023004814/wm5678sup3.pdf

CCDC references: 2246127, 2246128

Additional supporting information:  crystallographic information; 3D view; checkCIF report

## Figures and Tables

**Figure 4:**
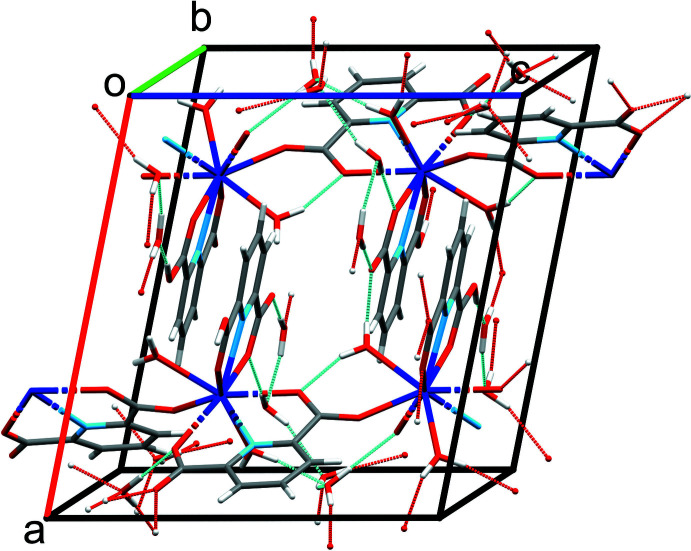
Hydrogen-bonding network in the [Sm(DPA)(HDPA)(H_2_O)_2_]·4H_2_O unit cell. Color code: Sm = dark blue, N = light blue, C = gray, H = white, and O = red.

**Table 1:** Hydrogen-bond geometry (Å, °) for Na_3_[Sm(DPA)_3_]·14H_2_O[Chem scheme1]

*D*—H⋯*A*	*D*—H	H⋯*A*	*D*⋯*A*	*D*—H⋯*A*
O1*W*—H1*WB*⋯O13*W* ^i^	0.85 (1)	1.98 (1)	2.8163 (19)	167 (3)
O2*W*—H2*WA*⋯O11*W* ^ii^	0.86 (1)	2.10 (1)	2.927 (2)	160 (3)
O2*W*—H2*WA*⋯O12*W* ^iii^	0.86 (1)	2.76 (3)	3.2317 (19)	116 (2)
O2*W*—H2*WB*⋯O1*W*	0.86 (1)	1.90 (1)	2.742 (2)	164 (3)
O4*W*—H4*WA*⋯O5*W*	0.85 (1)	1.91 (1)	2.741 (2)	167 (3)
O4*W*—H4*WB*⋯O5	0.85 (1)	2.02 (1)	2.8534 (17)	169 (3)
O6*W*—H6*WA*⋯O12^iv^	0.86 (1)	2.00 (1)	2.8435 (19)	168 (3)
O6*W*—H6*WB*⋯O8^v^	0.87 (1)	2.09 (2)	2.8645 (18)	149 (3)
O7*W*—H7*WA*⋯O6*W*	0.84 (1)	1.93 (1)	2.7611 (19)	167 (3)
O7*W*—H7*WB*⋯O3^vi^	0.85 (1)	2.54 (2)	3.1551 (17)	131 (2)
O7*W*—H7*WB*⋯O5^vi^	0.85 (1)	2.31 (2)	3.0267 (17)	142 (2)
O8*W*—H8*WA*⋯O10^vii^	0.84 (1)	1.87 (1)	2.7065 (17)	171 (2)
O9*W*—H9*WA*⋯O6^vi^	0.85 (1)	1.94 (1)	2.7871 (16)	175 (2)
O9*W*—H9*WB*⋯O1^viii^	0.85 (1)	1.87 (1)	2.7160 (16)	173 (2)
O10*W*—H10*A*⋯O2*W* ^viii^	0.85 (1)	1.90 (1)	2.7419 (18)	178 (2)
O11*W*—H11*A*⋯O9	0.85 (1)	1.97 (1)	2.8128 (17)	174 (3)
O11*W*—H11*B*⋯O12*W*	0.85 (1)	2.13 (1)	2.973 (2)	178 (3)
O12*W*—H12*A*⋯O2^iii^	0.85 (1)	2.11 (1)	2.9542 (18)	173 (3)
O12*W*—H12*B*⋯O8*W* ^vii^	0.86 (1)	2.03 (2)	2.8057 (18)	149 (3)
O13*W*—H13*A*⋯O12*W* ^vii^	0.85 (1)	1.99 (1)	2.8235 (18)	170 (2)
O13*W*—H13*B*⋯O7^viii^	0.85 (1)	2.09 (1)	2.9274 (17)	171 (2)
O13*W*—H13*B*⋯O8^viii^	0.85 (1)	2.57 (2)	3.1929 (17)	132 (2)

Symmetry codes: (i) 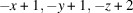 ; (ii) 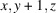 ; (iii) 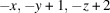 ; (iv) 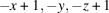 ; (v) 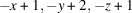 ; (vi) 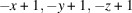 ; (vii) 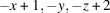 ; (viii) 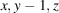 .

**Table 2:** Hydrogen-bond geometry (Å, °) for [Sm(DPA)(HDPA)(H_2_O)_2_]·4H_2_O[Chem scheme1]

*D*—H⋯*A*	*D*—H	H⋯*A*	*D*⋯*A*	*D*—H⋯*A*
O1*W*—H1*WA*⋯O2^i^	0.85 (1)	1.88 (1)	2.7218 (16)	169 (2)
O1*W*—H1*WB*⋯O7	0.85 (1)	1.91 (1)	2.7103 (15)	157 (2)
O2*W*—H2*WA*⋯O3*W*	0.85 (1)	1.87 (1)	2.7115 (16)	170 (2)
O2*W*—H2*WB*⋯O6*W* ^ii^	0.85 (1)	1.90 (1)	2.7193 (16)	163 (2)
O3*W*—H3*WA*⋯O5^iii^	0.85 (1)	2.04 (1)	2.8679 (15)	164 (2)
O3*W*—H3*WB*⋯O6^iv^	0.85 (1)	2.01 (1)	2.8568 (16)	173 (2)
O4*W*—H4*WA*⋯O2^v^	0.85 (1)	1.79 (1)	2.6360 (18)	174 (3)
O4*W*—H4*WB*⋯O5*W*	0.85 (1)	2.05 (2)	2.773 (2)	143 (3)
O5*W*—H5*WA*⋯O1^v^	0.85 (1)	2.10 (1)	2.9340 (17)	167 (2)
O5*W*—H5*WB*⋯O6*W* ^vi^	0.85 (1)	2.14 (1)	2.9579 (19)	162 (2)
O6*W*—H6*WA*⋯O3*W* ^vii^	0.85 (1)	2.10 (1)	2.8696 (16)	151 (2)
O6*W*—H6*WB*⋯O6	0.85 (1)	1.83 (1)	2.6828 (16)	177 (2)
O4—H4⋯O4*W*	1.01 (3)	1.47 (3)	2.4703 (19)	174 (2)

Symmetry codes: (i) 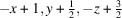 ; (ii) 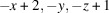 ; (iii) 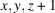 ; (iv) 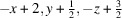 ; (v) 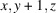 ; (vi) 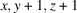 ; (vii) 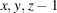 .

**Table 3:** Experimental details

	Na_3_[Sm(C_7_H_3_NO_4_)_3_]·14H_2_O	[Sm(C_7_H_3_NO_4_)(C_7_H_4_NO_4_)(H_2_O)_2_]·4H_2_O
Crystal data		
*M*_r_	966.85	589.66
Crystal system, space group	Triclinic, *P* 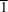	Monoclinic, *P*2_1_/*c*
Temperature (K)	100	100
*a*, *b*, *c* (Å)	10.2674 (10), 10.9688 (10), 17.1570 (16)	13.9292 (8), 11.1969 (7), 12.8086 (7)
α, β, γ (°)	73.835 (3), 77.573 (3), 72.894 (3)	90, 103.049 (2), 90
*V* (Å^3^)	1754.9 (3)	1946.1 (2)
*Z*	2	4
Radiation type	Mo *K*α	Mo *K*α
μ (mm^−1^)	1.81	3.10
Crystal size (mm)	0.78 × 0.58 × 0.26	0.48 × 0.40 × 0.15

Data collection		
Diffractometer	Bruker APEXII CCD	Bruker APEXII CCD
Absorption correction	Multi-scan (*SADABS*; Bruker, 2019 ▸)	Multi-scan (*SADABS*; Bruker, 2019 ▸)
*T*_min_, *T*_max_	0.615, 0.747	0.575, 0.747
No. of measured, independent and observed [*I* > 2σ(*I*)] reflections	107477, 13465, 12700	74345, 7424, 6679
*R*_int_	0.045	0.044
(sin θ/λ)_max_ (Å^−1^)	0.771	0.769

Refinement		
*R*[*F* ^2^ > 2σ(*F* ^2^)], *wR*(*F* ^2^), *S*	0.023, 0.058, 1.11	0.018, 0.042, 1.08
No. of reflections	13465	7424
No. of parameters	574	319
No. of restraints	28	12
H-atom treatment	H atoms treated by a mixture of independent and constrained refinement	H atoms treated by a mixture of independent and constrained refinement
Δρ_max_, Δρ_min_ (e Å^−3^)	3.32, −1.15	0.73, −1.09

Computer programs: *APEX2* and *SAINT* (Bruker, 2019 ▸), *SHELXT* (Sheldrick, 2015*a* ▸), *SHELXL* (Sheldrick, 2015*b* ▸), *OLEX2* (Dolomanov *et al.*, 2009 ▸) and *publCIF* (Westrip, 2010 ▸).

## supplementary crystallographic information

### Trisodium tris(pyridine-2,6-dicarboxylato-κ3O2,N,O6)samarate(III) tetradecahydrate (I) Crystal data

**Table:**

Na_3_[Sm(C_7_H_3_NO_4_)_3_]·14H_2_O	*Z* = 2
*M**_r_* = 966.85	*F*(000) = 974
Triclinic, *P*1	*D*_x_ = 1.830 Mg m^−^^3^
*a* = 10.2674 (10) Å	Mo *K*α radiation, λ = 0.71073 Å
*b* = 10.9688 (10) Å	Cell parameters from 9982 reflections
*c* = 17.1570 (16) Å	θ = 2.4–33.2°
α = 73.835 (3)°	µ = 1.81 mm^−^^1^
β = 77.573 (3)°	*T* = 100 K
γ = 72.894 (3)°	Prism, colourless
*V* = 1754.9 (3) Å^3^	0.78 × 0.58 × 0.26 mm

### Trisodium tris(pyridine-2,6-dicarboxylato-κ3O2,N,O6)samarate(III) tetradecahydrate (I) Data collection

**Table:**

Bruker APEXII CCD diffractometer	12700 reflections with *I* > 2σ(*I*)
φ and ω scans	*R*_int_ = 0.045
Absorption correction: multi-scan (SADABS; Bruker, 2019)	θ_max_ = 33.2°, θ_min_ = 2.0°
*T*_min_ = 0.615, *T*_max_ = 0.747	*h* = −15→15
107477 measured reflections	*k* = −16→16
13465 independent reflections	*l* = −26→26

### Trisodium tris(pyridine-2,6-dicarboxylato-κ3O2,N,O6)samarate(III) tetradecahydrate (I) Refinement

**Table:**

Refinement on *F*^2^	28 restraints
Least-squares matrix: full	Hydrogen site location: mixed
*R*[*F*^2^ > 2σ(*F*^2^)] = 0.023	H atoms treated by a mixture of independent and constrained refinement
*wR*(*F*^2^) = 0.058	*w* = 1/[σ^2^(*F*_o_^2^) + (0.021*P*)^2^ + 2.2772*P*] where *P* = (*F*_o_^2^ + 2*F*_c_^2^)/3
*S* = 1.11	(Δ/σ)_max_ = 0.004
13465 reflections	Δρ_max_ = 3.32 e Å^−^^3^
574 parameters	Δρ_min_ = −1.15 e Å^−^^3^

### Trisodium tris(pyridine-2,6-dicarboxylato-κ3O2,N,O6)samarate(III) tetradecahydrate (I) Special details

**Table:**

Geometry. All esds (except the esd in the dihedral angle between two l.s. planes) are estimated using the full covariance matrix. The cell esds are taken into account individually in the estimation of esds in distances, angles and torsion angles; correlations between esds in cell parameters are only used when they are defined by crystal symmetry. An approximate (isotropic) treatment of cell esds is used for estimating esds involving l.s. planes.

### Trisodium tris(pyridine-2,6-dicarboxylato-κ3O2,N,O6)samarate(III) tetradecahydrate (I) Fractional atomic coordinates and isotropic or equivalent isotropic displacement parameters (Å^2^)

**Table:**

	*x*	*y*	*z*	*U*_iso_*/*U*_eq_	
Sm1	0.48815 (2)	0.74844 (2)	0.75088 (2)	0.00321 (2)	
Na1	0.500000	0.500000	0.500000	0.00980 (16)	
Na2	0.50358 (6)	0.25158 (6)	0.75459 (4)	0.00837 (11)	
Na3	0.14568 (7)	1.30026 (7)	0.74024 (5)	0.01388 (13)	
Na4	0.500000	0.000000	1.000000	0.00854 (16)	
O1	0.32228 (11)	0.96519 (11)	0.72600 (7)	0.00735 (19)	
O2	0.10717 (12)	1.09223 (11)	0.74353 (8)	0.0111 (2)	
O3	0.42909 (11)	0.53805 (11)	0.78804 (7)	0.00775 (19)	
O4	0.29239 (12)	0.41204 (11)	0.78682 (8)	0.0117 (2)	
O5	0.47032 (11)	0.71148 (11)	0.62035 (7)	0.00748 (19)	
O6	0.55858 (13)	0.70498 (12)	0.49004 (7)	0.0109 (2)	
O7	0.60619 (11)	0.90222 (11)	0.77079 (7)	0.00738 (19)	
O8	0.69830 (11)	1.07466 (11)	0.72233 (7)	0.00862 (19)	
O9	0.39263 (11)	−0.21216 (11)	0.88660 (7)	0.00830 (19)	
O10	0.43084 (12)	−0.19347 (12)	1.00559 (7)	0.0103 (2)	
O11	0.70788 (11)	−0.41510 (11)	0.71542 (7)	0.00843 (19)	
O12	0.89428 (11)	−0.57662 (11)	0.74960 (7)	0.0100 (2)	
N1	0.23451 (12)	0.75259 (12)	0.76118 (8)	0.0053 (2)	
N2	0.60933 (13)	0.87570 (12)	0.62274 (8)	0.0055 (2)	
N3	0.63346 (13)	−0.37902 (12)	0.86490 (8)	0.0059 (2)	
C1	0.19098 (15)	0.98546 (14)	0.73974 (9)	0.0061 (2)	
C2	0.13830 (14)	0.86619 (14)	0.75074 (9)	0.0065 (2)	
C3	0.00166 (16)	0.87325 (16)	0.74916 (11)	0.0123 (3)	
H3	−0.063565	0.953392	0.742608	0.015*	
C4	−0.03531 (17)	0.75749 (17)	0.75761 (13)	0.0169 (3)	
H4	−0.125743	0.759170	0.755871	0.020*	
C5	0.06437 (16)	0.63935 (16)	0.76868 (12)	0.0132 (3)	
H5	0.042040	0.560674	0.774643	0.016*	
C6	0.19804 (14)	0.64151 (14)	0.77064 (9)	0.0065 (2)	
C7	0.31407 (15)	0.51924 (14)	0.78274 (9)	0.0066 (2)	
C8	0.54421 (15)	0.74740 (14)	0.55205 (9)	0.0065 (2)	
C9	0.61918 (15)	0.84837 (14)	0.55005 (9)	0.0063 (2)	
C10	0.69509 (17)	0.90667 (16)	0.47973 (10)	0.0110 (3)	
H10	0.702967	0.884285	0.430172	0.013*	
C11	0.75889 (19)	0.99887 (18)	0.48485 (10)	0.0141 (3)	
H11	0.809417	1.039876	0.438477	0.017*	
C12	0.74666 (17)	1.02945 (16)	0.55983 (10)	0.0114 (3)	
H12	0.787242	1.092062	0.564500	0.014*	
C13	0.67220 (15)	0.96399 (14)	0.62765 (9)	0.0062 (2)	
C14	0.65829 (14)	0.98425 (14)	0.71282 (9)	0.0059 (2)	
C15	0.46267 (15)	−0.24388 (14)	0.94523 (9)	0.0068 (2)	
C16	0.59566 (15)	−0.34874 (15)	0.93826 (9)	0.0072 (2)	
C17	0.67366 (17)	−0.40708 (17)	1.00180 (10)	0.0134 (3)	
H17	0.646641	−0.382103	1.051571	0.016*	
C18	0.79362 (18)	−0.50416 (19)	0.98895 (11)	0.0167 (3)	
H18	0.847093	−0.546644	1.030714	0.020*	
C19	0.83254 (16)	−0.53684 (17)	0.91333 (10)	0.0122 (3)	
H19	0.911577	−0.602159	0.903756	0.015*	
C20	0.75090 (15)	−0.46988 (14)	0.85216 (9)	0.0066 (2)	
C21	0.78861 (15)	−0.49090 (14)	0.76585 (9)	0.0068 (2)	
O1W	0.04883 (15)	1.24786 (15)	1.03199 (9)	0.0206 (3)	
H1WA	−0.008 (2)	1.201 (2)	1.0422 (18)	0.031*	
H1WB	0.1197 (19)	1.203 (2)	1.0544 (16)	0.031*	
O2W	0.14195 (14)	1.18981 (14)	0.88110 (9)	0.0173 (2)	
H2WA	0.122 (3)	1.1150 (15)	0.8944 (17)	0.026*	
H2WB	0.114 (3)	1.223 (3)	0.9238 (11)	0.026*	
O3W	0.13440 (13)	1.42018 (13)	0.60105 (9)	0.0175 (3)	
H3WA	0.190 (2)	1.467 (2)	0.5968 (17)	0.026*	
H3WB	0.0554 (15)	1.470 (2)	0.5944 (17)	0.026*	
O4W	0.28586 (13)	0.60772 (13)	0.57011 (8)	0.0153 (2)	
H4WA	0.221 (2)	0.665 (2)	0.5476 (15)	0.023*	
H4WB	0.331 (2)	0.644 (2)	0.5892 (15)	0.023*	
O5W	0.05492 (16)	0.76417 (16)	0.50456 (10)	0.0248 (3)	
H5WA	−0.011 (2)	0.728 (3)	0.5275 (18)	0.037*	
H5WB	0.078 (3)	0.751 (3)	0.4552 (9)	0.037*	
O6W	0.16147 (15)	0.72091 (14)	0.34836 (9)	0.0194 (3)	
H6WA	0.133 (3)	0.686 (3)	0.3180 (15)	0.029*	
H6WB	0.179 (3)	0.7923 (17)	0.3158 (14)	0.029*	
O7W	0.42934 (13)	0.58120 (12)	0.36593 (8)	0.0119 (2)	
H7WA	0.3439 (11)	0.614 (2)	0.3659 (16)	0.018*	
H7WB	0.444 (3)	0.5135 (17)	0.3476 (15)	0.018*	
O8W	0.62364 (12)	0.31062 (11)	0.83406 (7)	0.0097 (2)	
H8WA	0.597 (2)	0.276 (2)	0.8833 (7)	0.015*	
H8WB	0.569 (2)	0.3851 (13)	0.8228 (15)	0.015*	
O9W	0.37670 (12)	0.20510 (11)	0.67876 (7)	0.00911 (19)	
H9WA	0.391 (2)	0.233 (2)	0.6273 (6)	0.014*	
H9WB	0.365 (2)	0.1282 (13)	0.6909 (15)	0.014*	
O10W	0.42198 (12)	0.09077 (12)	0.86809 (7)	0.0103 (2)	
H10A	0.3359 (10)	0.122 (2)	0.8710 (15)	0.016*	
H10B	0.442 (3)	0.0245 (17)	0.8480 (14)	0.016*	
O11W	0.12847 (13)	−0.08265 (14)	0.95098 (9)	0.0170 (2)	
H11A	0.2100 (14)	−0.117 (3)	0.9312 (16)	0.025*	
H11B	0.132 (3)	−0.091 (3)	1.0010 (7)	0.025*	
O12W	0.13612 (13)	−0.11638 (14)	1.12813 (9)	0.0168 (2)	
H12A	0.0624 (18)	−0.111 (3)	1.1623 (13)	0.025*	
H12B	0.187 (2)	−0.1918 (15)	1.1490 (16)	0.025*	
O13W	0.71107 (12)	−0.07398 (12)	0.91019 (7)	0.0118 (2)	
H13A	0.751 (2)	−0.0124 (18)	0.8938 (15)	0.018*	
H13B	0.688 (3)	−0.077 (2)	0.8664 (10)	0.018*	
O14W	0.87868 (14)	−0.40551 (14)	0.56379 (8)	0.0173 (2)	
H14A	0.820 (2)	−0.413 (3)	0.6072 (11)	0.026*	
H14B	0.849 (3)	−0.412 (3)	0.5230 (12)	0.026*	

### Trisodium tris(pyridine-2,6-dicarboxylato-κ3O2,N,O6)samarate(III) tetradecahydrate (I) Atomic displacement parameters (Å^2^)

**Table:**

	*U*^11^	*U*^22^	*U*^33^	*U*^12^	*U*^13^	*U*^23^
Sm1	0.00305 (3)	0.00300 (3)	0.00357 (3)	−0.00045 (2)	−0.00041 (2)	−0.00111 (2)
Na1	0.0114 (4)	0.0097 (4)	0.0094 (4)	−0.0041 (3)	−0.0013 (3)	−0.0026 (3)
Na2	0.0084 (3)	0.0083 (3)	0.0095 (3)	−0.0017 (2)	−0.0021 (2)	−0.0035 (2)
Na3	0.0141 (3)	0.0118 (3)	0.0153 (3)	−0.0031 (2)	−0.0016 (3)	−0.0031 (3)
Na4	0.0096 (4)	0.0086 (4)	0.0074 (4)	−0.0014 (3)	−0.0015 (3)	−0.0024 (3)
O1	0.0053 (4)	0.0053 (4)	0.0107 (5)	−0.0013 (3)	−0.0009 (4)	−0.0009 (4)
O2	0.0087 (5)	0.0051 (5)	0.0181 (6)	0.0005 (4)	−0.0007 (4)	−0.0038 (4)
O3	0.0057 (4)	0.0051 (4)	0.0116 (5)	−0.0010 (3)	−0.0013 (4)	−0.0008 (4)
O4	0.0104 (5)	0.0048 (5)	0.0199 (6)	−0.0029 (4)	0.0004 (4)	−0.0039 (4)
O5	0.0089 (4)	0.0099 (5)	0.0054 (4)	−0.0053 (4)	0.0004 (4)	−0.0025 (4)
O6	0.0172 (5)	0.0126 (5)	0.0061 (5)	−0.0080 (4)	0.0004 (4)	−0.0041 (4)
O7	0.0092 (5)	0.0075 (5)	0.0064 (5)	−0.0036 (4)	−0.0016 (4)	−0.0013 (4)
O8	0.0089 (5)	0.0072 (5)	0.0121 (5)	−0.0031 (4)	−0.0019 (4)	−0.0044 (4)
O9	0.0072 (4)	0.0106 (5)	0.0060 (5)	0.0009 (4)	−0.0017 (4)	−0.0031 (4)
O10	0.0138 (5)	0.0098 (5)	0.0069 (5)	−0.0004 (4)	−0.0012 (4)	−0.0043 (4)
O11	0.0069 (4)	0.0097 (5)	0.0071 (5)	0.0006 (4)	−0.0012 (4)	−0.0020 (4)
O12	0.0064 (4)	0.0098 (5)	0.0127 (5)	0.0014 (4)	−0.0005 (4)	−0.0052 (4)
N1	0.0051 (5)	0.0045 (5)	0.0060 (5)	−0.0007 (4)	−0.0003 (4)	−0.0014 (4)
N2	0.0059 (5)	0.0047 (5)	0.0061 (5)	−0.0016 (4)	−0.0009 (4)	−0.0015 (4)
N3	0.0060 (5)	0.0049 (5)	0.0064 (5)	−0.0010 (4)	−0.0007 (4)	−0.0014 (4)
C1	0.0065 (6)	0.0045 (6)	0.0070 (6)	−0.0012 (4)	−0.0011 (4)	−0.0008 (5)
C2	0.0053 (5)	0.0054 (6)	0.0085 (6)	−0.0009 (4)	−0.0007 (4)	−0.0017 (5)
C3	0.0053 (6)	0.0076 (6)	0.0239 (8)	0.0002 (5)	−0.0031 (5)	−0.0048 (6)
C4	0.0070 (6)	0.0100 (7)	0.0358 (10)	−0.0024 (5)	−0.0046 (6)	−0.0071 (7)
C5	0.0068 (6)	0.0081 (7)	0.0260 (9)	−0.0025 (5)	−0.0019 (6)	−0.0057 (6)
C6	0.0054 (5)	0.0050 (6)	0.0092 (6)	−0.0015 (4)	0.0006 (4)	−0.0028 (5)
C7	0.0059 (5)	0.0052 (6)	0.0079 (6)	−0.0008 (4)	0.0006 (4)	−0.0018 (5)
C8	0.0075 (6)	0.0065 (6)	0.0060 (6)	−0.0022 (5)	−0.0012 (4)	−0.0015 (5)
C9	0.0074 (6)	0.0062 (6)	0.0056 (6)	−0.0023 (5)	−0.0011 (4)	−0.0010 (5)
C10	0.0148 (7)	0.0134 (7)	0.0070 (6)	−0.0086 (6)	0.0001 (5)	−0.0018 (5)
C11	0.0204 (8)	0.0174 (8)	0.0077 (7)	−0.0131 (6)	0.0019 (6)	−0.0017 (6)
C12	0.0151 (7)	0.0125 (7)	0.0093 (6)	−0.0099 (6)	0.0002 (5)	−0.0016 (5)
C13	0.0067 (5)	0.0054 (6)	0.0070 (6)	−0.0022 (4)	−0.0013 (4)	−0.0011 (5)
C14	0.0037 (5)	0.0055 (6)	0.0091 (6)	−0.0001 (4)	−0.0022 (4)	−0.0026 (5)
C15	0.0079 (6)	0.0062 (6)	0.0054 (6)	−0.0018 (5)	0.0003 (4)	−0.0010 (5)
C16	0.0074 (6)	0.0071 (6)	0.0067 (6)	−0.0004 (5)	−0.0022 (5)	−0.0014 (5)
C17	0.0128 (7)	0.0162 (7)	0.0085 (7)	0.0035 (6)	−0.0050 (5)	−0.0033 (6)
C18	0.0136 (7)	0.0210 (8)	0.0108 (7)	0.0062 (6)	−0.0072 (6)	−0.0033 (6)
C19	0.0091 (6)	0.0129 (7)	0.0110 (7)	0.0038 (5)	−0.0041 (5)	−0.0023 (5)
C20	0.0061 (5)	0.0057 (6)	0.0073 (6)	−0.0007 (4)	−0.0015 (4)	−0.0011 (5)
C21	0.0051 (5)	0.0067 (6)	0.0087 (6)	−0.0016 (4)	0.0003 (5)	−0.0029 (5)
O1W	0.0154 (6)	0.0214 (7)	0.0224 (7)	0.0007 (5)	−0.0086 (5)	−0.0020 (5)
O2W	0.0145 (6)	0.0194 (6)	0.0200 (6)	−0.0046 (5)	−0.0026 (5)	−0.0076 (5)
O3W	0.0104 (5)	0.0145 (6)	0.0238 (7)	−0.0024 (4)	0.0002 (5)	−0.0012 (5)
O4W	0.0120 (5)	0.0174 (6)	0.0193 (6)	−0.0056 (5)	−0.0020 (4)	−0.0070 (5)
O5W	0.0208 (7)	0.0254 (8)	0.0269 (8)	−0.0074 (6)	−0.0052 (6)	−0.0008 (6)
O6W	0.0188 (6)	0.0178 (6)	0.0235 (7)	−0.0049 (5)	−0.0067 (5)	−0.0045 (5)
O7W	0.0143 (5)	0.0099 (5)	0.0121 (5)	−0.0025 (4)	−0.0024 (4)	−0.0040 (4)
O8W	0.0101 (5)	0.0073 (5)	0.0109 (5)	−0.0004 (4)	−0.0035 (4)	−0.0010 (4)
O9W	0.0127 (5)	0.0082 (5)	0.0078 (5)	−0.0047 (4)	−0.0021 (4)	−0.0014 (4)
O10W	0.0105 (5)	0.0090 (5)	0.0102 (5)	0.0008 (4)	−0.0025 (4)	−0.0027 (4)
O11W	0.0093 (5)	0.0195 (6)	0.0188 (6)	0.0020 (5)	−0.0015 (5)	−0.0056 (5)
O12W	0.0098 (5)	0.0183 (6)	0.0216 (7)	−0.0027 (5)	0.0017 (5)	−0.0076 (5)
O13W	0.0107 (5)	0.0146 (6)	0.0096 (5)	−0.0023 (4)	−0.0026 (4)	−0.0023 (4)
O14W	0.0132 (6)	0.0215 (6)	0.0118 (6)	−0.0012 (5)	−0.0001 (4)	−0.0002 (5)

### Trisodium tris(pyridine-2,6-dicarboxylato-κ3O2,N,O6)samarate(III) tetradecahydrate (I) Geometric parameters (Å, º)

**Table:**

Sm1—O1	2.4700 (11)	N3—C20	1.3400 (19)
Sm1—O3	2.4367 (11)	C1—C2	1.508 (2)
Sm1—O5	2.4371 (11)	C2—C3	1.388 (2)
Sm1—O7	2.4764 (11)	C3—H3	0.9300
Sm1—O9^i^	2.4288 (11)	C3—C4	1.391 (2)
Sm1—O11^i^	2.5128 (11)	C4—H4	0.9300
Sm1—N1	2.5597 (12)	C4—C5	1.389 (2)
Sm1—N2	2.5428 (12)	C5—H5	0.9300
Sm1—N3^i^	2.5522 (13)	C5—C6	1.387 (2)
Na1—O6^ii^	2.4461 (12)	C6—C7	1.507 (2)
Na1—O6	2.4461 (12)	C8—C9	1.513 (2)
Na1—O4W	2.4087 (13)	C9—C10	1.389 (2)
Na1—O4W^ii^	2.4087 (13)	C10—H10	0.9300
Na1—H4WB	2.54 (3)	C10—C11	1.387 (2)
Na1—O7W^ii^	2.4100 (13)	C11—H11	0.9300
Na1—O7W	2.4100 (13)	C11—C12	1.389 (2)
Na2—Na3^iii^	3.6071 (10)	C12—H12	0.9300
Na2—O4	2.4261 (13)	C12—C13	1.389 (2)
Na2—O8^iii^	2.4308 (13)	C13—C14	1.509 (2)
Na2—C7	3.1086 (16)	C15—C16	1.511 (2)
Na2—C14^iii^	3.0944 (16)	C16—C17	1.387 (2)
Na2—O7W^ii^	2.4764 (14)	C17—H17	0.9300
Na2—O8W	2.3415 (13)	C17—C18	1.393 (2)
Na2—H8WB	2.41 (2)	C18—H18	0.9300
Na2—O9W	2.2670 (13)	C18—C19	1.387 (2)
Na2—O10W	2.4208 (14)	C19—H19	0.9300
Na3—O2	2.4117 (14)	C19—C20	1.389 (2)
Na3—O4^i^	2.5614 (14)	C20—C21	1.512 (2)
Na3—O12^iv^	2.5306 (13)	O1W—H1WA	0.852 (10)
Na3—O2W	2.3805 (16)	O1W—H1WB	0.850 (10)
Na3—O3W	2.3931 (16)	O2W—H2WA	0.861 (10)
Na3—H3WA	2.66 (3)	O2W—H2WB	0.862 (10)
Na3—O9W^i^	2.4307 (14)	O3W—H3WA	0.847 (10)
Na4—O10	2.4001 (12)	O3W—H3WB	0.846 (10)
Na4—O10^v^	2.4001 (12)	O4W—H4WA	0.849 (10)
Na4—O10W^v^	2.4107 (12)	O4W—H4WB	0.847 (10)
Na4—O10W	2.4107 (12)	O5W—H5WA	0.852 (10)
Na4—O13W^v^	2.4400 (12)	O5W—H5WB	0.871 (10)
Na4—O13W	2.4400 (12)	O6W—H6WA	0.862 (10)
O1—C1	1.2800 (17)	O6W—H6WB	0.867 (10)
O2—C1	1.2416 (18)	O7W—H7WA	0.844 (10)
O3—C7	1.2828 (17)	O7W—H7WB	0.847 (10)
O4—C7	1.2404 (18)	O8W—H8WA	0.844 (9)
O5—C8	1.2806 (18)	O8W—H8WB	0.844 (9)
O6—C8	1.2401 (18)	O9W—H9WA	0.846 (9)
O7—C14	1.2810 (18)	O9W—H9WB	0.848 (9)
O8—C14	1.2399 (17)	O10W—H10A	0.845 (10)
O9—C15	1.2724 (18)	O10W—H10B	0.843 (10)
O10—C15	1.2458 (18)	O11W—H11A	0.848 (10)
O11—C21	1.2766 (18)	O11W—H11B	0.846 (10)
O12—C21	1.2468 (18)	O12W—H12A	0.849 (10)
N1—C2	1.3372 (19)	O12W—H12B	0.862 (10)
N1—C6	1.3354 (18)	O13W—H13A	0.845 (10)
N2—C9	1.3386 (19)	O13W—H13B	0.846 (10)
N2—C13	1.3409 (18)	O14W—H14A	0.852 (10)
N3—C16	1.3387 (19)	O14W—H14B	0.846 (10)
			
O1—Sm1—O7	74.91 (4)	O10—Na4—O13W^v^	90.20 (4)
O1—Sm1—O11^i^	153.30 (4)	O10W^v^—Na4—O10W	180.0
O1—Sm1—N1	62.60 (4)	O10W—Na4—O13W	79.58 (4)
O1—Sm1—N2	77.31 (4)	O10W—Na4—O13W^v^	100.42 (4)
O1—Sm1—N3^i^	133.62 (4)	O10W^v^—Na4—O13W^v^	79.58 (4)
O3—Sm1—O1	125.28 (4)	O10W^v^—Na4—O13W	100.42 (4)
O3—Sm1—O5	75.87 (4)	O13W^v^—Na4—O13W	180.0
O3—Sm1—O7	152.08 (4)	C1—O1—Sm1	125.52 (9)
O3—Sm1—O11^i^	74.52 (4)	C1—O2—Na3	130.03 (10)
O3—Sm1—N1	62.70 (4)	C7—O3—Sm1	126.16 (9)
O3—Sm1—N2	134.63 (4)	Na2—O4—Na3^iii^	92.60 (5)
O3—Sm1—N3^i^	78.41 (4)	C7—O4—Na2	111.84 (10)
O5—Sm1—O1	92.07 (4)	C7—O4—Na3^iii^	144.16 (11)
O5—Sm1—O7	126.55 (4)	C8—O5—Sm1	124.80 (9)
O5—Sm1—O11^i^	74.58 (4)	C8—O6—Na1	120.67 (10)
O5—Sm1—N1	75.42 (4)	C14—O7—Sm1	124.81 (9)
O5—Sm1—N2	63.44 (4)	C14—O8—Na2^i^	110.69 (9)
O5—Sm1—N3^i^	134.19 (4)	C15—O9—Sm1^iii^	123.86 (10)
O7—Sm1—O11^i^	94.32 (4)	C15—O10—Na4	120.46 (10)
O7—Sm1—N1	133.37 (4)	C21—O11—Sm1^iii^	125.58 (10)
O7—Sm1—N2	63.14 (4)	C21—O12—Na3^vi^	157.96 (10)
O7—Sm1—N3^i^	73.78 (4)	C2—N1—Sm1	120.78 (9)
O9^i^—Sm1—O1	75.25 (4)	C6—N1—Sm1	120.08 (9)
O9^i^—Sm1—O3	91.81 (4)	C6—N1—C2	118.93 (12)
O9^i^—Sm1—O5	152.88 (4)	C9—N2—Sm1	119.93 (9)
O9^i^—Sm1—O7	73.85 (4)	C9—N2—C13	119.10 (13)
O9^i^—Sm1—O11^i^	125.88 (4)	C13—N2—Sm1	120.81 (10)
O9^i^—Sm1—N1	77.46 (4)	C16—N3—Sm1^iii^	118.99 (10)
O9^i^—Sm1—N2	133.52 (4)	C16—N3—C20	118.90 (13)
O9^i^—Sm1—N3^i^	63.72 (4)	C20—N3—Sm1^iii^	121.98 (10)
O11^i^—Sm1—N1	132.31 (4)	O1—C1—C2	114.80 (13)
O11^i^—Sm1—N2	76.04 (4)	O2—C1—O1	125.97 (14)
O11^i^—Sm1—N3^i^	62.28 (4)	O2—C1—C2	119.22 (13)
N2—Sm1—N1	120.44 (4)	N1—C2—C1	114.50 (12)
N2—Sm1—N3^i^	116.16 (4)	N1—C2—C3	122.49 (14)
N3^i^—Sm1—N1	123.40 (4)	C3—C2—C1	123.00 (13)
O6^ii^—Na1—O6	180.00 (5)	C2—C3—H3	120.8
O6^ii^—Na1—H4WB	113.8 (4)	C2—C3—C4	118.35 (14)
O6—Na1—H4WB	66.2 (4)	C4—C3—H3	120.8
O4W^ii^—Na1—O6	97.97 (4)	C3—C4—H4	120.4
O4W—Na1—O6^ii^	97.97 (4)	C5—C4—C3	119.25 (15)
O4W^ii^—Na1—O6^ii^	82.03 (4)	C5—C4—H4	120.4
O4W—Na1—O6	82.03 (4)	C4—C5—H5	120.8
O4W—Na1—O4W^ii^	180.0	C6—C5—C4	118.42 (14)
O4W—Na1—H4WB	19.5 (3)	C6—C5—H5	120.8
O4W^ii^—Na1—H4WB	160.5 (3)	N1—C6—C5	122.55 (14)
O4W^ii^—Na1—O7W	85.01 (4)	N1—C6—C7	114.55 (12)
O4W—Na1—O7W^ii^	85.01 (4)	C5—C6—C7	122.90 (13)
O4W—Na1—O7W	94.99 (4)	O3—C7—Na2	81.93 (8)
O4W^ii^—Na1—O7W^ii^	94.99 (4)	O3—C7—C6	114.75 (12)
O7W^ii^—Na1—O6^ii^	91.13 (4)	O4—C7—Na2	46.42 (8)
O7W^ii^—Na1—O6	88.87 (4)	O4—C7—O3	125.91 (14)
O7W—Na1—O6	91.13 (4)	O4—C7—C6	119.34 (13)
O7W—Na1—O6^ii^	88.87 (4)	C6—C7—Na2	157.60 (10)
O7W^ii^—Na1—H4WB	74.3 (5)	O5—C8—C9	115.43 (13)
O7W—Na1—H4WB	105.7 (5)	O6—C8—O5	125.08 (14)
O7W—Na1—O7W^ii^	180.0	O6—C8—C9	119.48 (13)
Na3^iii^—Na2—H8WB	119.8 (4)	N2—C9—C8	114.36 (12)
O4—Na2—Na3^iii^	45.18 (3)	N2—C9—C10	121.99 (13)
O4—Na2—O8^iii^	173.35 (5)	C10—C9—C8	123.64 (13)
O4—Na2—C7	21.74 (4)	C9—C10—H10	120.6
O4—Na2—C14^iii^	151.35 (4)	C11—C10—C9	118.75 (15)
O4—Na2—O7W^ii^	89.10 (5)	C11—C10—H10	120.6
O4—Na2—H8WB	75.0 (4)	C10—C11—H11	120.3
O8^iii^—Na2—Na3^iii^	128.45 (4)	C10—C11—C12	119.43 (15)
O8^iii^—Na2—C7	164.79 (4)	C12—C11—H11	120.3
O8^iii^—Na2—C14^iii^	22.02 (4)	C11—C12—H12	120.9
O8^iii^—Na2—O7W^ii^	94.67 (5)	C13—C12—C11	118.19 (14)
O8^iii^—Na2—H8WB	111.1 (4)	C13—C12—H12	120.9
C7—Na2—Na3^iii^	65.15 (3)	N2—C13—C12	122.50 (14)
C7—Na2—H8WB	57.5 (3)	N2—C13—C14	114.75 (12)
C14^iii^—Na2—Na3^iii^	106.82 (3)	C12—C13—C14	122.74 (13)
C14^iii^—Na2—C7	171.39 (4)	O7—C14—Na2^i^	103.23 (9)
C14^iii^—Na2—H8WB	131.1 (3)	O7—C14—C13	115.40 (12)
O7W^ii^—Na2—Na3^iii^	101.31 (4)	O8—C14—Na2^i^	47.30 (7)
O7W^ii^—Na2—C7	74.28 (4)	O8—C14—O7	125.03 (14)
O7W^ii^—Na2—C14^iii^	105.37 (5)	O8—C14—C13	119.55 (13)
O7W^ii^—Na2—H8WB	80.2 (5)	C13—C14—Na2^i^	121.13 (9)
O8W—Na2—Na3^iii^	135.37 (4)	O9—C15—C16	116.08 (13)
O8W—Na2—O4	92.78 (5)	O10—C15—O9	124.98 (14)
O8W—Na2—O8^iii^	92.68 (5)	O10—C15—C16	118.92 (13)
O8W—Na2—C7	77.19 (4)	N3—C16—C15	114.28 (13)
O8W—Na2—C14^iii^	111.41 (4)	N3—C16—C17	122.66 (14)
O8W—Na2—O7W^ii^	90.09 (5)	C17—C16—C15	123.05 (14)
O8W—Na2—H8WB	20.4 (3)	C16—C17—H17	120.9
O8W—Na2—O10W	93.71 (5)	C16—C17—C18	118.15 (15)
O9W—Na2—Na3^iii^	41.54 (3)	C18—C17—H17	120.9
O9W—Na2—O4	83.79 (5)	C17—C18—H18	120.3
O9W—Na2—O8^iii^	90.77 (5)	C19—C18—C17	119.40 (15)
O9W—Na2—C7	99.36 (5)	C19—C18—H18	120.3
O9W—Na2—C14^iii^	72.04 (4)	C18—C19—H19	120.7
O9W—Na2—O7W^ii^	89.31 (5)	C18—C19—C20	118.56 (15)
O9W—Na2—O8W	176.53 (5)	C20—C19—H19	120.7
O9W—Na2—H8WB	156.3 (3)	N3—C20—C19	122.26 (14)
O9W—Na2—O10W	86.76 (5)	N3—C20—C21	114.72 (12)
O10W—Na2—Na3^iii^	74.40 (3)	C19—C20—C21	123.01 (13)
O10W—Na2—O4	88.49 (5)	O11—C21—C20	115.27 (13)
O10W—Na2—O8^iii^	87.38 (4)	O12—C21—O11	126.00 (14)
O10W—Na2—C7	104.39 (4)	O12—C21—C20	118.71 (13)
O10W—Na2—C14^iii^	75.29 (4)	H1WA—O1W—H1WB	109 (3)
O10W—Na2—O7W^ii^	175.59 (5)	Na3—O2W—H2WA	116.0 (19)
O10W—Na2—H8WB	102.7 (5)	Na3—O2W—H2WB	128.6 (19)
Na2^i^—Na3—H3WA	86.7 (4)	H2WA—O2W—H2WB	107 (3)
O2—Na3—Na2^i^	108.54 (4)	Na3—O3W—H3WA	99.2 (19)
O2—Na3—O4^i^	144.68 (5)	Na3—O3W—H3WB	112.4 (19)
O2—Na3—O12^iv^	95.66 (5)	H3WA—O3W—H3WB	109 (3)
O2—Na3—H3WA	119.8 (4)	Na1—O4W—H4WA	126.1 (18)
O2—Na3—O9W^i^	83.28 (4)	Na1—O4W—H4WB	88.8 (18)
O4^i^—Na3—Na2^i^	42.21 (3)	H4WA—O4W—H4WB	110 (3)
O4^i^—Na3—H3WA	83.5 (3)	H5WA—O5W—H5WB	107 (3)
O12^iv^—Na3—Na2^i^	154.56 (4)	H6WA—O6W—H6WB	104 (3)
O12^iv^—Na3—O4^i^	112.43 (5)	Na1—O7W—Na2^ii^	131.75 (5)
O12^iv^—Na3—H3WA	88.0 (6)	Na1—O7W—H7WA	114.8 (18)
O2W—Na3—Na2^i^	81.53 (4)	Na1—O7W—H7WB	104.9 (18)
O2W—Na3—O2	76.69 (5)	Na2^ii^—O7W—H7WA	94.6 (18)
O2W—Na3—O4^i^	78.94 (5)	Na2^ii^—O7W—H7WB	104.5 (18)
O2W—Na3—O12^iv^	97.00 (5)	H7WA—O7W—H7WB	103 (2)
O2W—Na3—O3W	176.16 (6)	Na2—O8W—H8WA	106.0 (17)
O2W—Na3—H3WA	162.4 (4)	Na2—O8W—H8WB	84.7 (17)
O2W—Na3—O9W^i^	103.12 (5)	H8WA—O8W—H8WB	106 (2)
O3W—Na3—Na2^i^	101.53 (4)	Na2—O9W—Na3^iii^	100.26 (5)
O3W—Na3—O2	104.30 (5)	Na2—O9W—H9WA	117.2 (17)
O3W—Na3—O4^i^	101.73 (5)	Na2—O9W—H9WB	118.1 (17)
O3W—Na3—O12^iv^	79.24 (5)	Na3^iii^—O9W—H9WA	113.2 (17)
O3W—Na3—H3WA	18.3 (3)	Na3^iii^—O9W—H9WB	94.1 (17)
O3W—Na3—O9W^i^	80.70 (5)	H9WA—O9W—H9WB	111 (2)
O9W^i^—Na3—Na2^i^	38.20 (3)	Na2—O10W—H10A	101.1 (17)
O9W^i^—Na3—O4^i^	77.79 (4)	Na2—O10W—H10B	102.4 (17)
O9W^i^—Na3—O12^iv^	158.99 (5)	Na4—O10W—Na2	128.07 (5)
O9W^i^—Na3—H3WA	74.6 (6)	Na4—O10W—H10A	113.7 (17)
O10^v^—Na4—O10	180.00 (6)	Na4—O10W—H10B	102.6 (17)
O10—Na4—O10W^v^	92.22 (4)	H10A—O10W—H10B	107 (2)
O10—Na4—O10W	87.78 (4)	H11A—O11W—H11B	105 (3)
O10^v^—Na4—O10W	92.22 (4)	H12A—O12W—H12B	103 (3)
O10^v^—Na4—O10W^v^	87.78 (4)	Na4—O13W—H13A	107.4 (17)
O10^v^—Na4—O13W^v^	89.80 (4)	Na4—O13W—H13B	107.5 (17)
O10—Na4—O13W	89.80 (4)	H13A—O13W—H13B	102 (2)
O10^v^—Na4—O13W	90.20 (4)	H14A—O14W—H14B	112 (3)
			
Sm1—O1—C1—O2	164.44 (12)	O9—C15—C16—C17	−170.69 (15)
Sm1—O1—C1—C2	−16.46 (18)	O10—C15—C16—N3	−168.08 (13)
Sm1—O3—C7—Na2	149.87 (9)	O10—C15—C16—C17	10.7 (2)
Sm1—O3—C7—O4	165.65 (12)	N1—C2—C3—C4	−0.9 (3)
Sm1—O3—C7—C6	−14.47 (18)	N1—C6—C7—Na2	−130.8 (2)
Sm1—O5—C8—O6	162.94 (12)	N1—C6—C7—O3	4.68 (19)
Sm1—O5—C8—C9	−16.30 (18)	N1—C6—C7—O4	−175.43 (14)
Sm1—O7—C14—Na2^i^	121.58 (8)	N2—C9—C10—C11	−2.0 (2)
Sm1—O7—C14—O8	168.27 (11)	N2—C13—C14—Na2^i^	−115.99 (12)
Sm1—O7—C14—C13	−12.81 (17)	N2—C13—C14—O7	9.65 (19)
Sm1^iii^—O9—C15—O10	157.66 (12)	N2—C13—C14—O8	−171.36 (13)
Sm1^iii^—O9—C15—C16	−20.84 (17)	N3—C16—C17—C18	−2.2 (3)
Sm1^iii^—O11—C21—O12	176.34 (11)	N3—C20—C21—O11	3.42 (19)
Sm1^iii^—O11—C21—C20	−5.05 (17)	N3—C20—C21—O12	−177.86 (13)
Sm1—N1—C2—C1	−4.62 (17)	C1—C2—C3—C4	178.19 (16)
Sm1—N1—C2—C3	174.58 (12)	C2—N1—C6—C5	1.1 (2)
Sm1—N1—C6—C5	−173.65 (12)	C2—N1—C6—C7	−179.45 (13)
Sm1—N1—C6—C7	5.79 (17)	C2—C3—C4—C5	1.1 (3)
Sm1—N2—C9—C8	4.58 (16)	C3—C4—C5—C6	−0.2 (3)
Sm1—N2—C9—C10	−174.00 (12)	C4—C5—C6—N1	−0.9 (3)
Sm1—N2—C13—C12	175.90 (12)	C4—C5—C6—C7	179.67 (16)
Sm1—N2—C13—C14	−2.79 (16)	C5—C6—C7—Na2	48.6 (3)
Sm1^iii^—N3—C16—C15	3.18 (16)	C5—C6—C7—O3	−175.88 (15)
Sm1^iii^—N3—C16—C17	−175.62 (12)	C5—C6—C7—O4	4.0 (2)
Sm1^iii^—N3—C20—C19	178.06 (12)	C6—N1—C2—C1	−179.35 (13)
Sm1^iii^—N3—C20—C21	−0.53 (16)	C6—N1—C2—C3	−0.1 (2)
Na1—O6—C8—O5	−17.7 (2)	C8—C9—C10—C11	179.54 (15)
Na1—O6—C8—C9	161.50 (10)	C9—N2—C13—C12	0.5 (2)
Na2—O4—C7—O3	−21.8 (2)	C9—N2—C13—C14	−178.15 (13)
Na2—O4—C7—C6	158.31 (11)	C9—C10—C11—C12	0.7 (3)
Na2^i^—O8—C14—O7	−74.53 (16)	C10—C11—C12—C13	1.1 (3)
Na2^i^—O8—C14—C13	106.58 (12)	C11—C12—C13—N2	−1.8 (2)
Na3—O2—C1—O1	−6.3 (2)	C11—C12—C13—C14	176.80 (15)
Na3—O2—C1—C2	174.60 (10)	C12—C13—C14—Na2^i^	65.32 (17)
Na3^iii^—O4—C7—Na2	−129.7 (2)	C12—C13—C14—O7	−169.04 (14)
Na3^iii^—O4—C7—O3	−151.47 (13)	C12—C13—C14—O8	10.0 (2)
Na3^iii^—O4—C7—C6	28.6 (3)	C13—N2—C9—C8	179.98 (13)
Na3^vi^—O12—C21—O11	125.3 (3)	C13—N2—C9—C10	1.4 (2)
Na3^vi^—O12—C21—C20	−53.3 (3)	C15—C16—C17—C18	179.08 (16)
Na4—O10—C15—O9	−87.53 (17)	C16—N3—C20—C19	2.1 (2)
Na4—O10—C15—C16	90.94 (14)	C16—N3—C20—C21	−176.44 (13)
O1—C1—C2—N1	12.93 (19)	C16—C17—C18—C19	1.5 (3)
O1—C1—C2—C3	−166.27 (15)	C17—C18—C19—C20	0.9 (3)
O2—C1—C2—N1	−167.90 (14)	C18—C19—C20—N3	−2.8 (2)
O2—C1—C2—C3	12.9 (2)	C18—C19—C20—C21	175.69 (15)
O5—C8—C9—N2	6.77 (19)	C19—C20—C21—O11	−175.16 (14)
O5—C8—C9—C10	−174.68 (15)	C19—C20—C21—O12	3.6 (2)
O6—C8—C9—N2	−172.51 (14)	C20—N3—C16—C15	179.22 (12)
O6—C8—C9—C10	6.0 (2)	C20—N3—C16—C17	0.4 (2)
O9—C15—C16—N3	10.52 (19)		

Symmetry codes: (i) *x*, *y*+1, *z*; (ii) −*x*+1, −*y*+1, −*z*+1; (iii) *x*, *y*−1, *z*; (iv) *x*−1, *y*+2, *z*; (v) −*x*+1, −*y*, −*z*+2; (vi) *x*+1, *y*−2, *z*.

### Trisodium tris(pyridine-2,6-dicarboxylato-κ3O2,N,O6)samarate(III) tetradecahydrate (I) Hydrogen-bond geometry (Å, º)

**Table:**

*D*—H···*A*	*D*—H	H···*A*	*D*···*A*	*D*—H···*A*
O1*W*—H1*WB*···O13*W*^vii^	0.85 (1)	1.98 (1)	2.8163 (19)	167 (3)
O2*W*—H2*WA*···O11*W*^i^	0.86 (1)	2.10 (1)	2.927 (2)	160 (3)
O2*W*—H2*WA*···O12*W*^viii^	0.86 (1)	2.76 (3)	3.2317 (19)	116 (2)
O2*W*—H2*WB*···O1*W*	0.86 (1)	1.90 (1)	2.742 (2)	164 (3)
O4*W*—H4*WA*···O5*W*	0.85 (1)	1.91 (1)	2.741 (2)	167 (3)
O4*W*—H4*WB*···O5	0.85 (1)	2.02 (1)	2.8534 (17)	169 (3)
O6*W*—H6*WA*···O12^ix^	0.86 (1)	2.00 (1)	2.8435 (19)	168 (3)
O6*W*—H6*WB*···O8^x^	0.87 (1)	2.09 (2)	2.8645 (18)	149 (3)
O7*W*—H7*WA*···O6*W*	0.84 (1)	1.93 (1)	2.7611 (19)	167 (3)
O7*W*—H7*WB*···O3^ii^	0.85 (1)	2.54 (2)	3.1551 (17)	131 (2)
O7*W*—H7*WB*···O5^ii^	0.85 (1)	2.31 (2)	3.0267 (17)	142 (2)
O8*W*—H8*WA*···O10^v^	0.84 (1)	1.87 (1)	2.7065 (17)	171 (2)
O9*W*—H9*WA*···O6^ii^	0.85 (1)	1.94 (1)	2.7871 (16)	175 (2)
O9*W*—H9*WB*···O1^iii^	0.85 (1)	1.87 (1)	2.7160 (16)	173 (2)
O10*W*—H10*A*···O2*W*^iii^	0.85 (1)	1.90 (1)	2.7419 (18)	178 (2)
O11*W*—H11*A*···O9	0.85 (1)	1.97 (1)	2.8128 (17)	174 (3)
O11*W*—H11*B*···O12*W*	0.85 (1)	2.13 (1)	2.973 (2)	178 (3)
O12*W*—H12*A*···O2^viii^	0.85 (1)	2.11 (1)	2.9542 (18)	173 (3)
O12*W*—H12*B*···O8*W*^v^	0.86 (1)	2.03 (2)	2.8057 (18)	149 (3)
O13*W*—H13*A*···O12*W*^v^	0.85 (1)	1.99 (1)	2.8235 (18)	170 (2)
O13*W*—H13*B*···O7^iii^	0.85 (1)	2.09 (1)	2.9274 (17)	171 (2)
O13*W*—H13*B*···O8^iii^	0.85 (1)	2.57 (2)	3.1929 (17)	132 (2)

Symmetry codes: (i) *x*, *y*+1, *z*; (ii) −*x*+1, −*y*+1, −*z*+1; (iii) *x*, *y*−1, *z*; (v) −*x*+1, −*y*, −*z*+2; (vii) −*x*+1, −*y*+1, −*z*+2; (viii) −*x*, −*y*+1, −*z*+2; (ix) −*x*+1, −*y*, −*z*+1; (x) −*x*+1, −*y*+2, −*z*+1.

### catena-Poly[[[diaqua(6-carboxypyridine-2-carboxylato-κ3O2,N,O6)samarium(III)]-µ-pyridine-2,6-dicarboxylato-κ4O2,N,O6:O2] tetrahydrate] (II) Crystal data

**Table:**

[Sm(C_7_H_3_NO_4_)(C_7_H_4_NO_4_)(H_2_O)_2_]·4H_2_O	*F*(000) = 1164
*M**_r_* = 589.66	*D*_x_ = 2.013 Mg m^−^^3^
Monoclinic, *P*2_1_/*c*	Mo *K*α radiation, λ = 0.71073 Å
*a* = 13.9292 (8) Å	Cell parameters from 9554 reflections
*b* = 11.1969 (7) Å	θ = 2.4–35.1°
*c* = 12.8086 (7) Å	µ = 3.10 mm^−^^1^
β = 103.049 (2)°	*T* = 100 K
*V* = 1946.1 (2) Å^3^	Plate, colourless
*Z* = 4	0.48 × 0.40 × 0.15 mm

### catena-Poly[[[diaqua(6-carboxypyridine-2-carboxylato-κ3O2,N,O6)samarium(III)]-µ-pyridine-2,6-dicarboxylato-κ4O2,N,O6:O2] tetrahydrate] (II) Data collection

**Table:**

Bruker APEXII CCD diffractometer	6679 reflections with *I* > 2σ(*I*)
φ and ω scans	*R*_int_ = 0.044
Absorption correction: multi-scan (SADABS; Bruker, 2019)	θ_max_ = 33.1°, θ_min_ = 2.4°
*T*_min_ = 0.575, *T*_max_ = 0.747	*h* = −21→21
74345 measured reflections	*k* = −17→17
7424 independent reflections	*l* = −17→19

### catena-Poly[[[diaqua(6-carboxypyridine-2-carboxylato-κ3O2,N,O6)samarium(III)]-µ-pyridine-2,6-dicarboxylato-κ4O2,N,O6:O2] tetrahydrate] (II) Refinement

**Table:**

Refinement on *F*^2^	12 restraints
Least-squares matrix: full	Hydrogen site location: mixed
*R*[*F*^2^ > 2σ(*F*^2^)] = 0.018	H atoms treated by a mixture of independent and constrained refinement
*wR*(*F*^2^) = 0.042	*w* = 1/[σ^2^(*F*_o_^2^) + (0.012*P*)^2^ + 2.2391*P*] where *P* = (*F*_o_^2^ + 2*F*_c_^2^)/3
*S* = 1.08	(Δ/σ)_max_ = 0.007
7424 reflections	Δρ_max_ = 0.73 e Å^−^^3^
319 parameters	Δρ_min_ = −1.09 e Å^−^^3^

### catena-Poly[[[diaqua(6-carboxypyridine-2-carboxylato-κ3O2,N,O6)samarium(III)]-µ-pyridine-2,6-dicarboxylato-κ4O2,N,O6:O2] tetrahydrate] (II) Special details

**Table:**

Geometry. All esds (except the esd in the dihedral angle between two l.s. planes) are estimated using the full covariance matrix. The cell esds are taken into account individually in the estimation of esds in distances, angles and torsion angles; correlations between esds in cell parameters are only used when they are defined by crystal symmetry. An approximate (isotropic) treatment of cell esds is used for estimating esds involving l.s. planes.

### catena-Poly[[[diaqua(6-carboxypyridine-2-carboxylato-κ3O2,N,O6)samarium(III)]-µ-pyridine-2,6-dicarboxylato-κ4O2,N,O6:O2] tetrahydrate] (II) Fractional atomic coordinates and isotropic or equivalent isotropic displacement parameters (Å^2^)

**Table:**

	*x*	*y*	*z*	*U*_iso_*/*U*_eq_	
Sm1	0.73193 (2)	0.29610 (2)	0.84878 (2)	0.00303 (2)	
O1	0.65850 (8)	0.10076 (9)	0.86514 (9)	0.00783 (19)	
O2	0.53228 (8)	−0.01891 (10)	0.87649 (10)	0.0123 (2)	
O3	0.64950 (8)	0.49371 (9)	0.85969 (9)	0.00847 (19)	
O4	0.51949 (9)	0.60513 (11)	0.87239 (10)	0.0145 (2)	
H4	0.5646 (18)	0.676 (2)	0.875 (2)	0.022*	
O5	0.84574 (8)	0.07310 (10)	0.27878 (9)	0.00810 (19)	
O6	0.92027 (9)	−0.10506 (10)	0.28114 (9)	0.0106 (2)	
O7	0.72787 (8)	0.22062 (9)	0.54309 (8)	0.00693 (18)	
O8	0.78295 (8)	0.16945 (10)	0.71580 (8)	0.00725 (18)	
C1	0.56995 (11)	0.08151 (13)	0.86995 (12)	0.0074 (2)	
C2	0.50502 (11)	0.18986 (13)	0.86725 (12)	0.0083 (2)	
C3	0.40535 (12)	0.18312 (16)	0.86702 (17)	0.0177 (3)	
H3	0.374223	0.108078	0.869860	0.021*	
C4	0.35229 (13)	0.28896 (17)	0.8625 (2)	0.0240 (4)	
H4A	0.283664	0.286989	0.860236	0.029*	
C5	0.40023 (12)	0.39746 (16)	0.86144 (16)	0.0176 (3)	
H5	0.365676	0.470880	0.859381	0.021*	
C6	0.50027 (11)	0.39530 (14)	0.86343 (12)	0.0091 (2)	
C7	0.56268 (11)	0.50434 (13)	0.86471 (12)	0.0083 (3)	
C8	0.88073 (10)	−0.02353 (13)	0.32337 (11)	0.0061 (2)	
C9	0.87488 (10)	−0.03865 (12)	0.43907 (11)	0.0052 (2)	
C10	0.90835 (11)	−0.13997 (13)	0.49895 (12)	0.0073 (2)	
H10	0.934576	−0.205672	0.467574	0.009*	
C11	0.90257 (11)	−0.14290 (13)	0.60575 (12)	0.0084 (2)	
H11	0.925058	−0.210990	0.648565	0.010*	
C12	0.86374 (10)	−0.04573 (13)	0.64965 (11)	0.0066 (2)	
H12	0.861422	−0.044590	0.723200	0.008*	
C13	0.82835 (10)	0.04988 (12)	0.58257 (11)	0.0049 (2)	
C14	0.77620 (10)	0.15538 (12)	0.61763 (11)	0.0049 (2)	
N1	0.55123 (9)	0.29383 (11)	0.86438 (10)	0.0063 (2)	
N2	0.83414 (9)	0.05330 (11)	0.47961 (9)	0.0046 (2)	
O1W	0.63395 (9)	0.34532 (11)	0.67167 (9)	0.0117 (2)	
H1WA	0.5864 (10)	0.3940 (15)	0.6529 (18)	0.018*	
H1WB	0.6530 (15)	0.3178 (19)	0.6179 (11)	0.018*	
O2W	0.87579 (8)	0.18700 (10)	0.93554 (9)	0.0091 (2)	
H2WA	0.8967 (16)	0.1939 (19)	1.0029 (3)	0.014*	
H2WB	0.9169 (11)	0.1431 (15)	0.9135 (16)	0.014*	
O3W	0.95920 (8)	0.19105 (10)	1.14835 (9)	0.00890 (19)	
H3WA	0.9230 (12)	0.1699 (19)	1.1904 (13)	0.013*	
H3WB	0.9949 (13)	0.2501 (12)	1.1748 (16)	0.013*	
O4W	0.63135 (11)	0.77889 (12)	0.89098 (16)	0.0307 (4)	
H4WA	0.6029 (18)	0.8463 (11)	0.888 (2)	0.046*	
H4WB	0.6931 (4)	0.785 (3)	0.896 (3)	0.046*	
O5W	0.80409 (10)	0.90705 (12)	0.90443 (12)	0.0217 (3)	
H5WA	0.7688 (15)	0.9695 (12)	0.900 (2)	0.033*	
H5WB	0.8408 (15)	0.919 (2)	0.9662 (9)	0.033*	
O6W	0.96769 (9)	−0.05672 (10)	0.09389 (9)	0.0116 (2)	
H6WA	0.9816 (16)	0.0173 (5)	0.0980 (18)	0.017*	
H6WB	0.9508 (15)	−0.072 (2)	0.1523 (9)	0.017*	

### catena-Poly[[[diaqua(6-carboxypyridine-2-carboxylato-κ3O2,N,O6)samarium(III)]-µ-pyridine-2,6-dicarboxylato-κ4O2,N,O6:O2] tetrahydrate] (II) Atomic displacement parameters (Å^2^)

**Table:**

	*U*^11^	*U*^22^	*U*^33^	*U*^12^	*U*^13^	*U*^23^
Sm1	0.00394 (3)	0.00300 (3)	0.00234 (3)	−0.00019 (2)	0.00113 (2)	−0.00035 (2)
O1	0.0069 (4)	0.0056 (4)	0.0115 (5)	−0.0009 (4)	0.0033 (4)	0.0000 (4)
O2	0.0093 (5)	0.0066 (5)	0.0214 (6)	−0.0034 (4)	0.0040 (4)	0.0004 (4)
O3	0.0085 (5)	0.0062 (4)	0.0105 (5)	0.0012 (4)	0.0017 (4)	−0.0005 (4)
O4	0.0147 (5)	0.0075 (5)	0.0220 (6)	0.0043 (4)	0.0055 (5)	−0.0020 (4)
O5	0.0116 (5)	0.0074 (4)	0.0064 (4)	0.0034 (4)	0.0044 (4)	0.0019 (4)
O6	0.0176 (5)	0.0080 (5)	0.0085 (5)	0.0054 (4)	0.0075 (4)	0.0001 (4)
O7	0.0088 (4)	0.0078 (5)	0.0043 (4)	0.0032 (4)	0.0018 (4)	0.0014 (3)
O8	0.0109 (5)	0.0079 (4)	0.0035 (4)	0.0020 (4)	0.0025 (4)	−0.0006 (4)
C1	0.0078 (6)	0.0066 (6)	0.0074 (6)	−0.0014 (5)	0.0010 (5)	−0.0009 (5)
C2	0.0064 (6)	0.0084 (6)	0.0109 (6)	0.0005 (5)	0.0032 (5)	0.0002 (5)
C3	0.0078 (6)	0.0134 (7)	0.0341 (10)	−0.0006 (5)	0.0090 (6)	0.0023 (7)
C4	0.0089 (7)	0.0171 (8)	0.0493 (13)	0.0024 (6)	0.0136 (8)	0.0039 (8)
C5	0.0113 (7)	0.0137 (7)	0.0301 (9)	0.0049 (6)	0.0095 (6)	0.0018 (7)
C6	0.0092 (6)	0.0083 (6)	0.0106 (6)	0.0025 (5)	0.0036 (5)	0.0005 (5)
C7	0.0112 (6)	0.0071 (6)	0.0070 (6)	0.0033 (5)	0.0025 (5)	−0.0001 (5)
C8	0.0080 (6)	0.0061 (6)	0.0053 (6)	0.0007 (4)	0.0034 (5)	−0.0003 (5)
C9	0.0060 (5)	0.0052 (5)	0.0049 (5)	0.0004 (4)	0.0022 (4)	0.0000 (4)
C10	0.0095 (6)	0.0058 (6)	0.0067 (6)	0.0021 (5)	0.0024 (5)	0.0000 (5)
C11	0.0117 (6)	0.0069 (6)	0.0066 (6)	0.0021 (5)	0.0017 (5)	0.0022 (5)
C12	0.0087 (6)	0.0064 (6)	0.0042 (6)	0.0004 (5)	0.0006 (5)	0.0002 (4)
C13	0.0060 (5)	0.0055 (5)	0.0033 (5)	−0.0003 (4)	0.0014 (4)	0.0001 (4)
C14	0.0057 (5)	0.0041 (5)	0.0051 (5)	−0.0007 (4)	0.0018 (4)	−0.0006 (4)
N1	0.0066 (5)	0.0069 (5)	0.0059 (5)	0.0008 (4)	0.0023 (4)	−0.0001 (4)
N2	0.0057 (5)	0.0044 (5)	0.0041 (5)	−0.0002 (4)	0.0023 (4)	−0.0003 (4)
O1W	0.0127 (5)	0.0169 (6)	0.0049 (5)	0.0090 (4)	0.0012 (4)	−0.0004 (4)
O2W	0.0088 (5)	0.0123 (5)	0.0055 (4)	0.0047 (4)	0.0003 (4)	−0.0017 (4)
O3W	0.0100 (5)	0.0109 (5)	0.0065 (5)	−0.0018 (4)	0.0032 (4)	−0.0005 (4)
O4W	0.0192 (7)	0.0093 (6)	0.0653 (12)	0.0012 (5)	0.0129 (7)	−0.0009 (6)
O5W	0.0188 (6)	0.0118 (6)	0.0316 (8)	0.0030 (5)	−0.0005 (5)	−0.0017 (5)
O6W	0.0169 (5)	0.0098 (5)	0.0107 (5)	0.0030 (4)	0.0087 (4)	0.0015 (4)

### catena-Poly[[[diaqua(6-carboxypyridine-2-carboxylato-κ3O2,N,O6)samarium(III)]-µ-pyridine-2,6-dicarboxylato-κ4O2,N,O6:O2] tetrahydrate] (II) Geometric parameters (Å, º)

**Table:**

Sm1—O2W	2.3952 (11)	C6—N1	1.3382 (19)
Sm1—O1W	2.4320 (11)	C6—C7	1.497 (2)
Sm1—O8	2.4422 (10)	C8—C9	1.512 (2)
Sm1—O1	2.4434 (11)	C9—N2	1.3360 (18)
Sm1—O5^i^	2.4707 (11)	C9—C10	1.3896 (19)
Sm1—O7^i^	2.5092 (11)	C10—C11	1.389 (2)
Sm1—O3	2.5112 (11)	C10—H10	0.9500
Sm1—N2^i^	2.5693 (12)	C11—C12	1.389 (2)
Sm1—N1	2.5695 (12)	C11—H11	0.9500
O1—C1	1.2673 (17)	C12—C13	1.3914 (19)
O2—C1	1.2516 (18)	C12—H12	0.9500
O3—C7	1.2313 (18)	C13—N2	1.3399 (18)
O4—C7	1.2930 (18)	C13—C14	1.507 (2)
O4—H4	1.01 (3)	O1W—H1WA	0.8499 (10)
O5—C8	1.2683 (17)	O1W—H1WB	0.8500 (10)
O6—C8	1.2505 (17)	O2W—H2WA	0.8500 (10)
O7—C14	1.2681 (17)	O2W—H2WB	0.8500 (10)
O8—C14	1.2495 (17)	O3W—H3WA	0.8499 (10)
C1—C2	1.509 (2)	O3W—H3WB	0.8499 (10)
C2—N1	1.3348 (19)	O4W—H4	1.47 (3)
C2—C3	1.390 (2)	O4W—H4WA	0.8499 (10)
C3—C4	1.391 (2)	O4W—H4WB	0.8499 (11)
C3—H3	0.9500	O5W—H5WA	0.8499 (10)
C4—C5	1.388 (3)	O5W—H5WB	0.8499 (10)
C4—H4A	0.9500	O6W—H6WA	0.8499 (10)
C5—C6	1.388 (2)	O6W—H6WB	0.8499 (10)
C5—H5	0.9500		
			
O2W—Sm1—O1W	141.52 (4)	C5—C4—C3	119.59 (16)
O2W—Sm1—O8	71.50 (4)	C5—C4—H4A	120.2
O1W—Sm1—O8	70.81 (4)	C3—C4—H4A	120.2
O2W—Sm1—O1	79.99 (4)	C4—C5—C6	117.89 (15)
O1W—Sm1—O1	97.19 (4)	C4—C5—H5	121.1
O8—Sm1—O1	74.54 (4)	C6—C5—H5	121.1
O2W—Sm1—O5^i^	86.13 (4)	N1—C6—C5	122.90 (15)
O1W—Sm1—O5^i^	78.35 (4)	N1—C6—C7	112.76 (13)
O8—Sm1—O5^i^	77.26 (4)	C5—C6—C7	124.34 (14)
O1—Sm1—O5^i^	151.28 (4)	O3—C7—O4	124.59 (14)
O2W—Sm1—O7^i^	72.88 (4)	O3—C7—C6	119.70 (13)
O1W—Sm1—O7^i^	143.96 (4)	O4—C7—C6	115.71 (13)
O8—Sm1—O7^i^	136.37 (4)	O6—C8—O5	126.15 (13)
O1—Sm1—O7^i^	75.20 (3)	O6—C8—C9	117.94 (13)
O5^i^—Sm1—O7^i^	124.35 (3)	O5—C8—C9	115.89 (12)
O2W—Sm1—O3	140.24 (4)	N2—C9—C10	122.25 (13)
O1W—Sm1—O3	71.63 (4)	N2—C9—C8	114.60 (12)
O8—Sm1—O3	139.39 (4)	C10—C9—C8	123.15 (12)
O1—Sm1—O3	125.34 (4)	C11—C10—C9	118.44 (13)
O5^i^—Sm1—O3	80.61 (4)	C11—C10—H10	120.8
O7^i^—Sm1—O3	84.12 (4)	C9—C10—H10	120.8
O2W—Sm1—N2^i^	75.51 (4)	C10—C11—C12	119.64 (13)
O1W—Sm1—N2^i^	124.84 (4)	C10—C11—H11	120.2
O8—Sm1—N2^i^	129.07 (4)	C12—C11—H11	120.2
O1—Sm1—N2^i^	135.45 (4)	C11—C12—C13	117.95 (13)
O5^i^—Sm1—N2^i^	62.69 (4)	C11—C12—H12	121.0
O7^i^—Sm1—N2^i^	62.31 (4)	C13—C12—H12	121.0
O3—Sm1—N2^i^	65.06 (4)	N2—C13—C12	122.55 (13)
O2W—Sm1—N1	133.67 (4)	N2—C13—C14	114.27 (12)
O1W—Sm1—N1	73.83 (4)	C12—C13—C14	123.12 (12)
O8—Sm1—N1	119.53 (4)	O8—C14—O7	126.27 (13)
O1—Sm1—N1	63.14 (4)	O8—C14—C13	117.83 (12)
O5^i^—Sm1—N1	138.95 (4)	O7—C14—C13	115.90 (12)
O7^i^—Sm1—N1	71.36 (4)	C2—N1—C6	118.86 (13)
O3—Sm1—N1	62.37 (4)	C2—N1—Sm1	119.84 (9)
N2^i^—Sm1—N1	111.35 (4)	C6—N1—Sm1	121.18 (10)
C1—O1—Sm1	125.96 (9)	C9—N2—C13	119.07 (12)
C7—O3—Sm1	123.70 (10)	C9—N2—Sm1^ii^	118.28 (9)
C7—O4—H4	113.1 (14)	C13—N2—Sm1^ii^	120.87 (9)
C8—O5—Sm1^ii^	123.71 (9)	Sm1—O1W—H1WA	130.3 (16)
C14—O7—Sm1^ii^	125.23 (9)	Sm1—O1W—H1WB	117.5 (15)
C14—O8—Sm1	143.85 (10)	H1WA—O1W—H1WB	112 (2)
O2—C1—O1	125.69 (14)	Sm1—O2W—H2WA	119.0 (15)
O2—C1—C2	117.75 (13)	Sm1—O2W—H2WB	134.2 (14)
O1—C1—C2	116.56 (13)	H2WA—O2W—H2WB	107 (2)
N1—C2—C3	122.34 (14)	H3WA—O3W—H3WB	110 (2)
N1—C2—C1	114.31 (12)	H4—O4W—H4WA	115 (2)
C3—C2—C1	123.35 (14)	H4—O4W—H4WB	132 (2)
C2—C3—C4	118.37 (16)	H4WA—O4W—H4WB	113 (3)
C2—C3—H3	120.8	H5WA—O5W—H5WB	99 (2)
C4—C3—H3	120.8	H6WA—O6W—H6WB	104 (2)
			
Sm1—O1—C1—O2	178.70 (11)	C10—C11—C12—C13	2.4 (2)
Sm1—O1—C1—C2	−1.16 (18)	C11—C12—C13—N2	−2.8 (2)
O2—C1—C2—N1	177.56 (14)	C11—C12—C13—C14	174.28 (13)
O1—C1—C2—N1	−2.6 (2)	Sm1—O8—C14—O7	−0.5 (3)
O2—C1—C2—C3	−2.8 (2)	Sm1—O8—C14—C13	−179.20 (10)
O1—C1—C2—C3	177.02 (16)	Sm1^ii^—O7—C14—O8	172.44 (11)
N1—C2—C3—C4	0.7 (3)	Sm1^ii^—O7—C14—C13	−8.81 (17)
C1—C2—C3—C4	−178.89 (18)	N2—C13—C14—O8	−167.61 (13)
C2—C3—C4—C5	−1.8 (3)	C12—C13—C14—O8	15.1 (2)
C3—C4—C5—C6	0.9 (3)	N2—C13—C14—O7	13.53 (18)
C4—C5—C6—N1	1.3 (3)	C12—C13—C14—O7	−163.75 (13)
C4—C5—C6—C7	−178.78 (18)	C3—C2—N1—C6	1.4 (2)
Sm1—O3—C7—O4	178.63 (11)	C1—C2—N1—C6	−179.02 (13)
Sm1—O3—C7—C6	−0.58 (19)	C3—C2—N1—Sm1	−174.76 (13)
N1—C6—C7—O3	4.5 (2)	C1—C2—N1—Sm1	4.83 (17)
C5—C6—C7—O3	−175.46 (16)	C5—C6—N1—C2	−2.4 (2)
N1—C6—C7—O4	−174.77 (13)	C7—C6—N1—C2	177.65 (13)
C5—C6—C7—O4	5.3 (2)	C5—C6—N1—Sm1	173.72 (13)
Sm1^ii^—O5—C8—O6	163.39 (12)	C7—C6—N1—Sm1	−6.26 (17)
Sm1^ii^—O5—C8—C9	−17.75 (17)	C10—C9—N2—C13	2.4 (2)
O6—C8—C9—N2	178.29 (13)	C8—C9—N2—C13	−178.01 (12)
O5—C8—C9—N2	−0.67 (19)	C10—C9—N2—Sm1^ii^	−162.53 (11)
O6—C8—C9—C10	−2.1 (2)	C8—C9—N2—Sm1^ii^	17.07 (15)
O5—C8—C9—C10	178.93 (13)	C12—C13—N2—C9	0.4 (2)
N2—C9—C10—C11	−2.6 (2)	C14—C13—N2—C9	−176.91 (12)
C8—C9—C10—C11	177.79 (13)	C12—C13—N2—Sm1^ii^	164.91 (10)
C9—C10—C11—C12	0.1 (2)	C14—C13—N2—Sm1^ii^	−12.39 (16)

Symmetry codes: (i) *x*, −*y*+1/2, *z*+1/2; (ii) *x*, −*y*+1/2, *z*−1/2.

### catena-Poly[[[diaqua(6-carboxypyridine-2-carboxylato-κ3O2,N,O6)samarium(III)]-µ-pyridine-2,6-dicarboxylato-κ4O2,N,O6:O2] tetrahydrate] (II) Hydrogen-bond geometry (Å, º)

**Table:**

*D*—H···*A*	*D*—H	H···*A*	*D*···*A*	*D*—H···*A*
O1*W*—H1*WA*···O2^iii^	0.85 (1)	1.88 (1)	2.7218 (16)	169 (2)
O1*W*—H1*WB*···O7	0.85 (1)	1.91 (1)	2.7103 (15)	157 (2)
O2*W*—H2*WA*···O3*W*	0.85 (1)	1.87 (1)	2.7115 (16)	170 (2)
O2*W*—H2*WB*···O6*W*^iv^	0.85 (1)	1.90 (1)	2.7193 (16)	163 (2)
O3*W*—H3*WA*···O5^v^	0.85 (1)	2.04 (1)	2.8679 (15)	164 (2)
O3*W*—H3*WB*···O6^vi^	0.85 (1)	2.01 (1)	2.8568 (16)	173 (2)
O4*W*—H4*WA*···O2^vii^	0.85 (1)	1.79 (1)	2.6360 (18)	174 (3)
O4*W*—H4*WB*···O5*W*	0.85 (1)	2.05 (2)	2.773 (2)	143 (3)
O5*W*—H5*WA*···O1^vii^	0.85 (1)	2.10 (1)	2.9340 (17)	167 (2)
O5*W*—H5*WB*···O6*W*^viii^	0.85 (1)	2.14 (1)	2.9579 (19)	162 (2)
O6*W*—H6*WA*···O3*W*^ix^	0.85 (1)	2.10 (1)	2.8696 (16)	151 (2)
O6*W*—H6*WB*···O6	0.85 (1)	1.83 (1)	2.6828 (16)	177 (2)
O4—H4···O4*W*	1.01 (3)	1.47 (3)	2.4703 (19)	174 (2)

Symmetry codes: (iii) −*x*+1, *y*+1/2, −*z*+3/2; (iv) −*x*+2, −*y*, −*z*+1; (v) *x*, *y*, *z*+1; (vi) −*x*+2, *y*+1/2, −*z*+3/2; (vii) *x*, *y*+1, *z*; (viii) *x*, *y*+1, *z*+1; (ix) *x*, *y*, *z*−1.

## References

[bb1] Aebischer, A., Gumy, F. & Bünzli, J. G. (2009). *Phys. Chem. Chem. Phys.* **11**, 1346–1353. 10.1039/b816131c 19224035

[bb2] Albertsson, J. (1970). *Acta Chem. Scand.* **24**, 3527–3541.

[bb3] Albertsson, J. (1972). *Acta Chem. Scand.* **26**, 985–1004.

[bb4] Brayshaw, P. A., Buenzli, J. G., Froidevaux, P., Harrowfield, J. M., Kim, Y. & Sobolev, A. N. (1995). *Inorg. Chem.* **34**, 2068–2076.

[bb5] Brayshaw, P. A., Hall, A. K., Harrison, W. T., Harrowfield, J. M., Pearce, D., Shand, T. M., Skelton, B. W., Whitaker, C. R. & White, A. H. (2005). *Eur. J. Inorg. Chem.* pp. 1127–1141.

[bb6] Bruker (2019). *APEX2*, *SAINT* and *SADABS*. Bruker AXS Inc., Madison, Wisconsin, USA.

[bb7] Carnall, W., Fields, P. & Rajnak, K. (1968). *J. Chem. Phys.* **49**, 4424–4442.

[bb8] Chauvin, A. S., Gumy, F., Imbert, D. & Bünzli, J. G. (2004). *Spectrosc. Lett.* **37**, 517–532.

[bb9] Cheisson, T. & Schelter, E. J. (2019). *Science*, **363**, 489–493. 10.1126/science.aau7628 30705185

[bb10] Chen, X., Jensen, M. & Liu, G. (2005). *J. Phys. Chem. B*, **109**, 13991–13999. 10.1021/jp0516700 16852756

[bb11] Cheng, C.-X., Liu, H.-W., Hu, Z.-Q., Luo, F.-H. & Cao, M.-N. (2007). *Acta Cryst.* E**63**, m1–m3.

[bb12] Chuasaard, T., Panyarat, K., Rodlamul, P., Chainok, K., Yimklan, S. & Rujiwatra, A. (2017). *Cryst. Growth Des.* **17**, 1045–1054.

[bb13] Dolomanov, O. V., Bourhis, L. J., Gildea, R. J., Howard, J. A. K. & Puschmann, H. (2009). *J. Appl. Cryst.* **42**, 339–341.

[bb14] Elahi, S. M. & Rajasekharan, M. V. (2016). *Chem. Sel.* **1**, 6515–6522.

[bb15] Eliseeva, S. V. & Bünzli, J. G. (2010). *Chem. Soc. Rev.* **39**, 189–227. 10.1039/b905604c 20023849

[bb16] Ghosh, S. K. & Bharadwaj, P. K. (2003). *Inorg. Chem.* **42**, 8250–8254. 10.1021/ic034976z 14658875

[bb17] Hojnik, N., Kristl, M., Golobič, A., Jagličić, Z. & Drofenik, M. (2015). *J. Mol. Struct.* **1079**, 54–60.

[bb18] Hou, K.-L., Bai, F.-Y., Xing, Y.-H., Cao, Y.-Z., Wei, D.-M. & Niu, S.-Y. (2011). *J. Inorg. Organomet. Polym.* **21**, 213–222.

[bb19] Hu, S., Dong, Z., Zhang, H. & Liu, Q. (1989). *J. Xiamen Univ.* **28**, 514–518.

[bb20] Judd, B. R. (1962). *Phys. Rev.* **127**, 750–761.

[bb21] Kang, S.-K. (2011). *Bull. Korean Chem. Soc.* **32**, 1745–1747.

[bb22] Kim, J.-G., Yoon, S.-K., Sohn, Y. & Kang, J.-G. (1998). *J. Alloys Compd.* **274**, 1–9.

[bb23] Kofod, N., Nawrocki, P., Juelsholt, M., Christiansen, T. L., Jensen, K. M. & Sørensen, T. J. (2020). *Inorg. Chem.* **59**, 10409–10421. 10.1021/acs.inorgchem.0c00056 32108485

[bb24] Kong, Y.-J., Hou, G.-Z., Gong, Z.-N., Zhao, F.-T. & Han, L.-J. (2022). *RSC Adv.* **12**, 8435–8442. 10.1039/d2ra00077f PMC8984937 35424814

[bb25] Kumar, D., Tewari, S., Adnan, M., Ahmad, S., Vijaya Prakash, G. & Ramanan, A. (2019). *Inorg. Chim. Acta*, **487**, 81–91.

[bb26] Li, Q.-F., Ge, G.-W., Sun, Y., Yu, M. & Wang, Z. (2019). *Spectrochim. Acta A Mol. Biomol. Spectrosc.* **214**, 333–338. 10.1016/j.saa.2019.02.056 30798215

[bb27] Liu, H. X., Liu, Q., Xu, Y., Huang, T. T., Wang, L. T., Ye, K. Q. & Zeng, G. (2014). *Adv. Mater. Res.* **834–836**, 490–493.

[bb28] Liu, S.-H., Li, Y.-Z. & Meng, Q.-J. (2005). *Acta Cryst.* E**61**, m1111–m1113.

[bb29] Lupei, A., Tiseanu, C., Gheorghe, C. & Voicu, F. (2012). *Appl. Phys. B*, **108**, 909–918.

[bb30] Moghzi, F., Soleimannejad, J., Emadi, H. & Janczak, J. (2020). *Acta Cryst.* B**76**, 779–788. 10.1107/S2052520620009336 33017311

[bb31] Mondry, A. & Starynowicz, P. (1995). *J. Alloys Compd.* **225**, 367–371.

[bb32] Mortensen, S. S., Marciniak Nielsen, M. A., Nawrocki, P. R. & Sørensen, T. J. (2022). *J. Phys. Chem. A*, **126**, 8596–8605. 10.1021/acs.jpca.2c04793 36367508

[bb33] Murray, G. M., Sarrio, R. V. & Peterson, J. R. (1990). *Inorg. Chim. Acta*, **176**, 233–240.

[bb34] Najafi, A., Mirzaei, M., Bauzá, A., Mague, J. T. & Frontera, A. (2017). *Inorg. Chem. Commun.* **83**, 24–26.

[bb35] Ofelt, G. (1962). *J. Chem. Phys.* **37**, 511–520.

[bb52] PicoQuant (2018). *EasyTau 2*. Picoquant, Germany. https://www.pico­quant.com

[bb36] Rafizadeh, M., Amani, V., Iravani, E. & Neumüller, B. (2005). *Z. Anorg. Allg. Chem.* **631**, 952–955.

[bb37] Sakirzanovas, S., Katelnikovas, A., Bettentrup, H., Kareiva, A. & Jüstel, T. (2011). *J. Lumin.* **131**, 1525–1529.

[bb38] Salaam, J., N’Dala–Louika, I., Balogh, C., Suleimanov, I., Pilet, G., Veyre, L., Camp, C., Thieuleux, C., Riobé, F. & Maury, O. (2022). *Eur. J. Inorg. Chem.* **2022**, 00412.

[bb39] Sharif, S., Khan, B., Şahin, O. & Khan, I. (2016). *Russ. J. Coord. Chem.* **42**, 56–65.

[bb40] Sheldrick, G. M. (2015*a*). *Acta Cryst.* A**71**, 3–8.

[bb41] Sheldrick, G. M. (2015*b*). *Acta Cryst.* C**71**, 3–8.

[bb42] Skaudzius, R., Sakirzanovas, S. & Kareiva, A. (2018). *J. Elec Materi*. **47**, 3951–3956.

[bb43] Song, Y.-S., Yan, B. & Chen, Z.-X. (2005). *J. Mol. Struct.* **750**, 101–108.

[bb44] Storm Thomsen, M., Anker, A. S., Kacenauskaite, L. & Sørensen, T. J. (2022). *Dalton Trans.* **51**, 8960–8963. 10.1039/d2dt01522f 35660819

[bb45] Tancrez, N., Feuvrie, C., Ledoux, I., Zyss, J., Toupet, L., Le Bozec, H. & Maury, O. (2005). *J. Am. Chem. Soc.* **127**, 13474–13475. 10.1021/ja054065j 16190692

[bb46] Viveros-Andrade, A. G., Colorado-Peralta, R., Flores-Alamo, M., Castillo-Blum, S. E., Durán-Hernández, J. & Rivera, J. M. (2017). *J. Mol. Struct.* **1145**, 10–17.

[bb47] Wang, P., Fan, R.-Q., Liu, X.-R., Yang, Y.-L. & Zhou, G.-P. (2012). *J. Inorg. Organomet. Polym.* **22**, 744–755.

[bb48] Westrip, S. P. (2010). *J. Appl. Cryst.* **43**, 920–925.

[bb49] Wybourne, B. G. (2004). *J. Alloys Compd.* **380**, 96–100.

[bb50] Xu, X., Liu, X., Sun, T., Zhang, X. & Wang, E. (2009). *J. Coord. Chem.* **62**, 2755–2763.

[bb51] Zhou, D., Huang, C., Wang, K. & Xu, G. (1994). *Polyhedron*, **13**, 987–991.

