# Supplementary material for: Crystal structures of two SmIII complexes with dipicolinate [DPA]2− ligands: comparison of luminescent properties of products obtained at different pH values
Source: Acta Crystallogr E Crystallogr Commun. 2023 Jun 9;79(Pt 7):619–25. doi: 10.1107/S2056989023004814 (PMC10439428; doi:10.1107/S2056989023004814)
Supplement: Supplementary file 4 [file e-79-00619-sup3.pdf]

Supporting Information

Crystal Structures of Two Sm^{3+}-Complexes with Dipicolinate $[DPA]^{3-}$ - Comparison of Luminescent Properties at Different pH

Sabina Svava Mortensen[†]*, and Thomas Just Sørensen**

Department of Chemistry & Nano-Science Center, University of Copenhagen, Universitetsparken 5, 2100 København Ø, Denmark. TJS@chem.ku.dk

This is the supporting information for the article, showing the additionally measured spectra, symmetry deviation analysis, and PXRD.

Table of Content

Table of Content ... 2
1 Symmetry deviation values ... 3
2 PXRD ... 5
2.1 Simulated PXRD for Single Crystals ... 5
2.2 PXRD for Powdered Crystals ... 6
3 Spectroscopy ... 7
3.1 Luminescence Lifetimes ... 7
3.1.1 Powdered Crystals Luminescence Lifetimes at Different pH ... 7
3.2 Sm(DPA) pH = 2 ... 8
3.2.1 Sm(DPA) pH = 2 Excitation Spectrum ... 8
3.2.2 Sm(DPA) pH = 2 Luminescence Lifetimes ... 9
3.3 Sm(DPA) pH = 5 ... 10
3.3.1 Sm(DPA) pH = 5 Excitation Spectrum ... 10
3.3.2 Sm(DPA) pH = 5 Luminescence Lifetimes ... 11
3.4 Sm(DPA) pH = 7 ... 12
3.4.1 Sm(DPA) pH = 7 Excitation Spectrum ... 12
3.4.2 Sm(DPA) pH = 7 Luminescence Lifetimes ... 13
3.5 Sm(DPA) pH = 10 ... 14
3.5.1 Sm(DPA) pH = 10 Excitation Spectrum ... 14
3.5.2 Sm(DPA) pH = 10 Luminescence Lifetimes ... 15
4 References ... 15

1 Symmetry deviation values

The central Sm^{3+} ion in $Na_3[Sm(DPA)_3]\cdot 14\ H_2O$ and $[Sm(DPA)(HDPA)(H_2O)_2]\cdot 4\ H_2O$ is both none-coordinated. For nine-coordinated systems the Trigonal Tricapped Prism (TTP) and the capped Square Antiprism (cSAP) are the two possible arrangements of ligand atoms around the Sm^{3+} ion with the lowest potential energy.(Thomson, 1904, Drew, 1977) To evaluate the site symmetry for the two EuDPA and deviation from the ideal TTP and cSAP structure, a symmetry deviation value was calculated.

This is achieved by the use of AlignIt developed by M. Storm Thomsen et al. AlignIt uses Eq. 1, after manual rotational optimization in Mercury, to calculate a symmetry deviation value, σ_{ideal}:(Thomsen *et al.*, 2022)

$$\sigma_{ideal}(P-Q)=\sum_{k=1}^{N}\frac{|Q_k-P_k|^2}{|Q_k-Q_O|^2}\cdot\frac{100}{N} \qquad \text{Eq. 1}$$

where *P* is a point in the ideal structure, and *Q* is a point in the distorted structure. Q_0 is the origin in the distorted model.(Thomsen *et al.*, 2022) For two identical structures, the symmetry deviation value will be zero, $\sigma_{ideal} = 0$.(Thomsen *et al.*, 2022)

Table 1 compares the $Na_3[Sm(DPA)_3]\cdot 14\ H_2O$ and $[Sm(DPA)(HDPA)(H_2O)_2]\cdot 4\ H_2O$ structures with different ideal structures to determine what ideal geometry describes each structure best and the degree of deviation. The two EuDPA complexes are compared to a range of different ideal geometries: The capped square antiprism (cSAP), and trigonal tricapped prism (TTP), both which are observed for nine-coordinated lanthanide(III) complexes The Hula hoop (HOOP), triangular cupola (TCup), and hexagonal bipyramid (HBPy) are used to create a scale for symmetry with larger deviation values. Muffin (MFF) is introduced as an in-between value for scale comparison. The scale ranges from 0 to 27, where $\sigma_{ideal} = 0$ corresponds to two identical structures, meaning there is no deviation.

Table 1: Symmetry deviation values, σ_{ideal}, for comparison of ideal nine-coordinated structures. Values were calculated using AlignIt.(Thomsen *et al.*, 2022)

	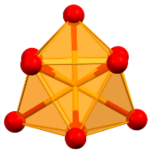	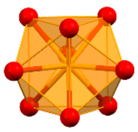	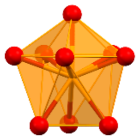	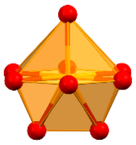	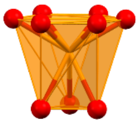	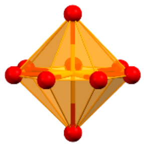
	**cSAP**	**TTP**	**MFF**	**HOOP**	**TCup**	**HBPy**
**cSAP**	0	0.97	13.51	20.15	16.71	26.83
**TTP**		0	9.63	14.12	19.26	23.25
**MUFF**			0	14.61	20.40	24.92
**HOOP**				0	19.79	14.49
**Tcup**					0	21.52
**HBPy**						0

Table 2: Symmetry deviation values, σ_{ideal}, for $Na_3[Sm(DPA)_3]\cdot 14\ H_2O$ and $[Sm(DPA)(HDPA)(H_2O)_2]\cdot 4\ H_2O$.

		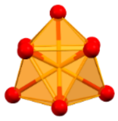	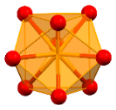	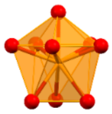	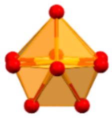	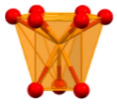	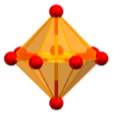
		**cSAP**	**TTP**	**MFF**	**HOOP**	**TCup**	**HBPy**
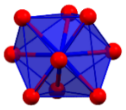	**$Na_3[Sm(DPA)_3]\cdot 14\ H_2O$**	1.80	**1.16**	10.37	16.27	19.38	24.84
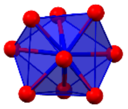	**$[Sm(DPA)(HDPA)(H_2O)_2]\cdot 4\ H_2O$**	2.42*	**0.73**	9.20	12.69	18.63	20.98

*This value might be lower if the structure was oriented in a different direction, where a different atom occupies the capping position.

2 PXRD

2.1 Simulated PXRD for Single Crystals

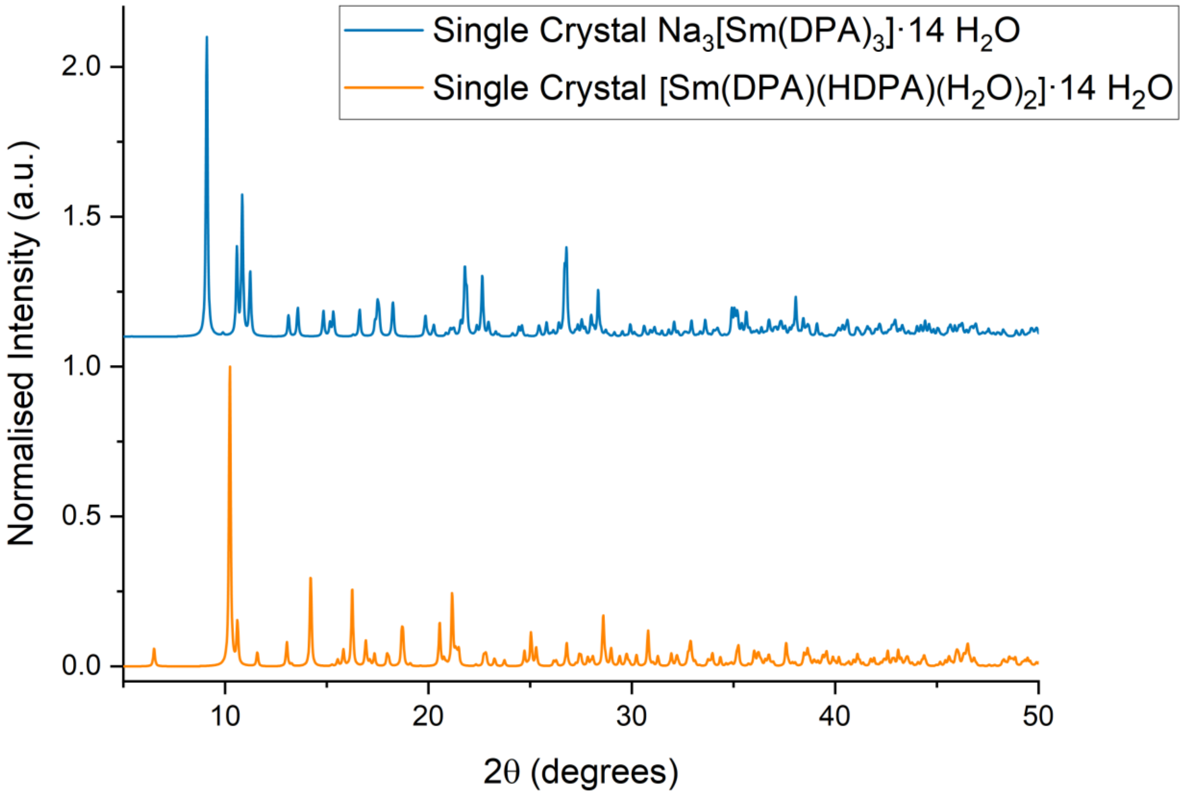

Figure 1: PXRD for $Na_3[Sm(DPA)_3]\cdot 14\ H_2O$ and $[Sm(DPA)(HDPA)(H_2O)_2]\cdot 4\ H_2O$ simulated from the CIF files.

2.2 PXRD for Powdered Crystals

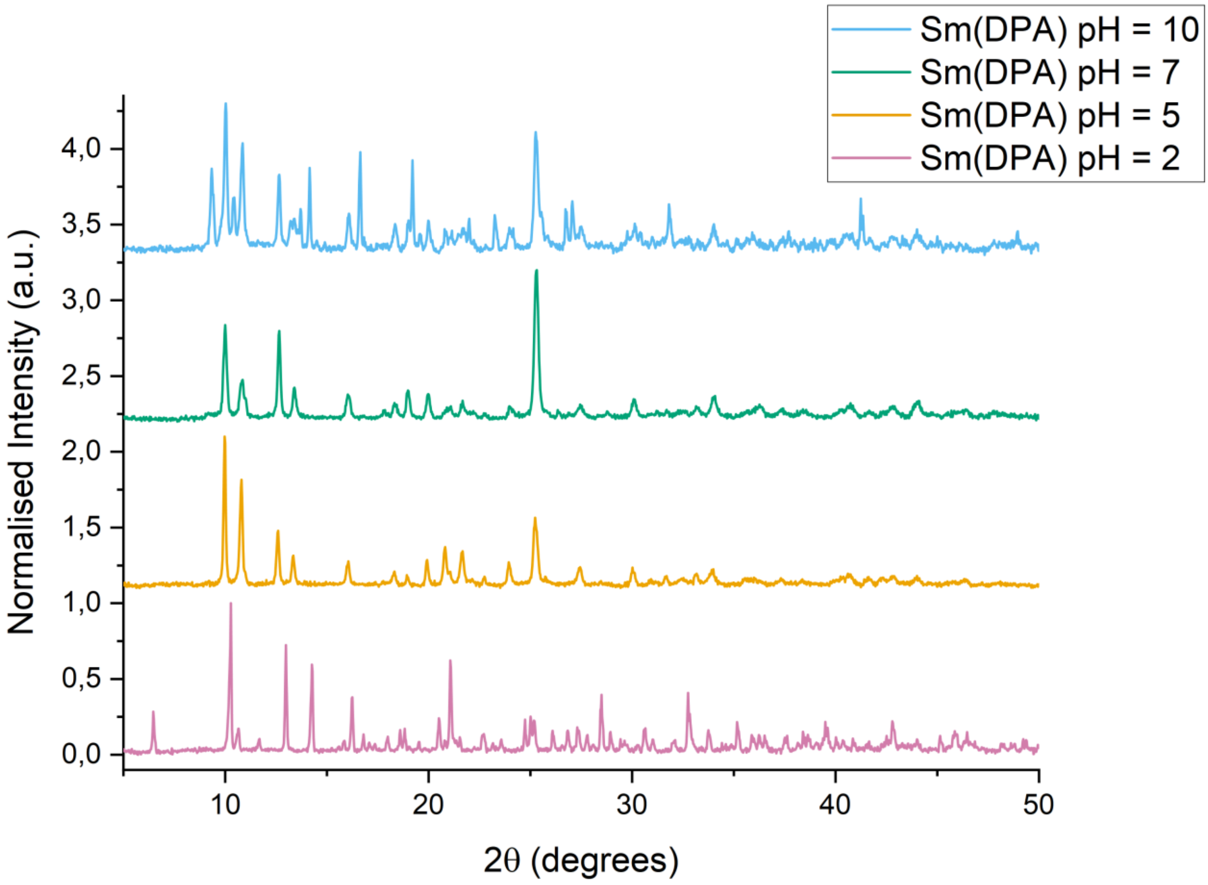

Figure 2: PXRD for the crystals crushed to a powder with samples at pH = 2, pH = 5, pH = 7, and pH = 10.

3 Spectroscopy

3.1 Luminescence Lifetimes

3.1.1 Powdered Crystals Luminescence Lifetimes at Different pH

Table 3: Luminescence lifetimes For Sm(DPA) at pH = 2, pH = 5, pH = 7, and pH = 10 in 2-methyltetrahydrofuran glass at 77 K.

Complex	τ_{obs} (µs)
Sm(DPA) pH = 2	8.893
Sm(DPA) pH = 5	28.589
Sm(DPA) pH = 7	28.417
Sm(DPA) pH = 10	28.298

3.2 Sm(DPA) pH = 2

3.2.1 Sm(DPA) pH = 2 Excitation Spectrum

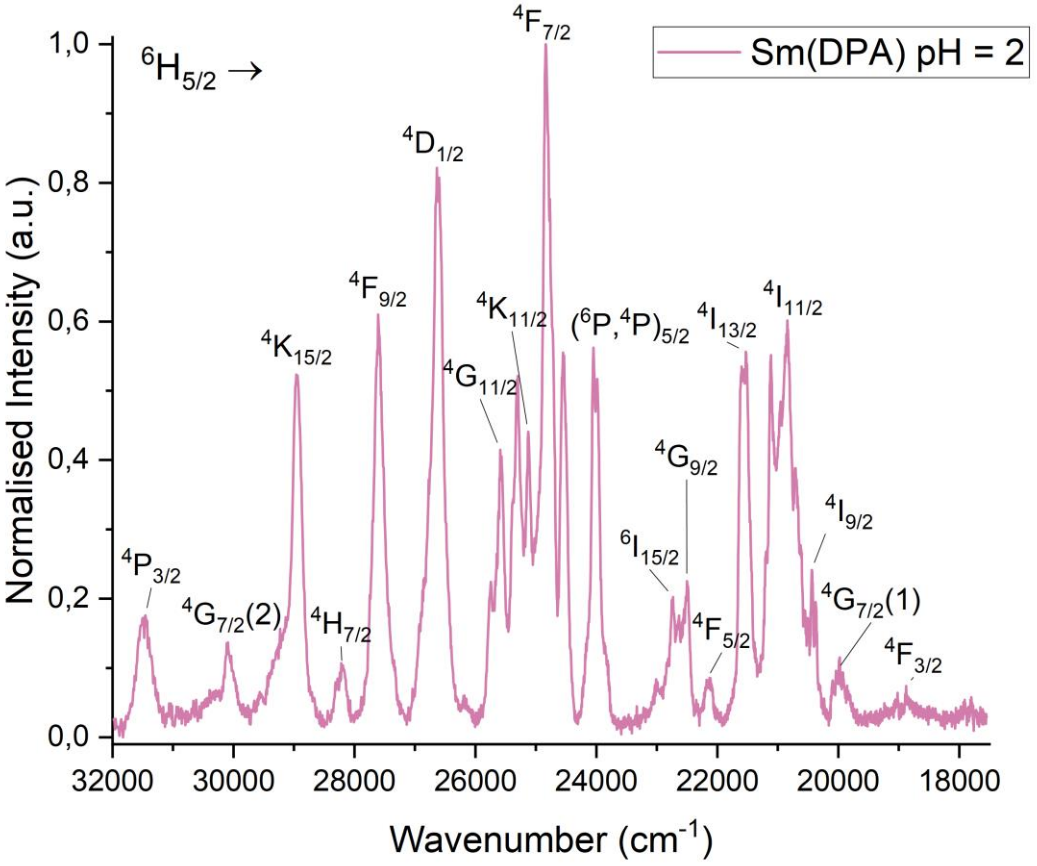

Figure 3: Normalised excitation spectra (emission at 614 nm) for Sm(DPA) pH = 2 in 2-methyltetrahydrofuran glass at 77 K.

3.2.2 Sm(DPA) pH = 2 Luminescence Lifetimes

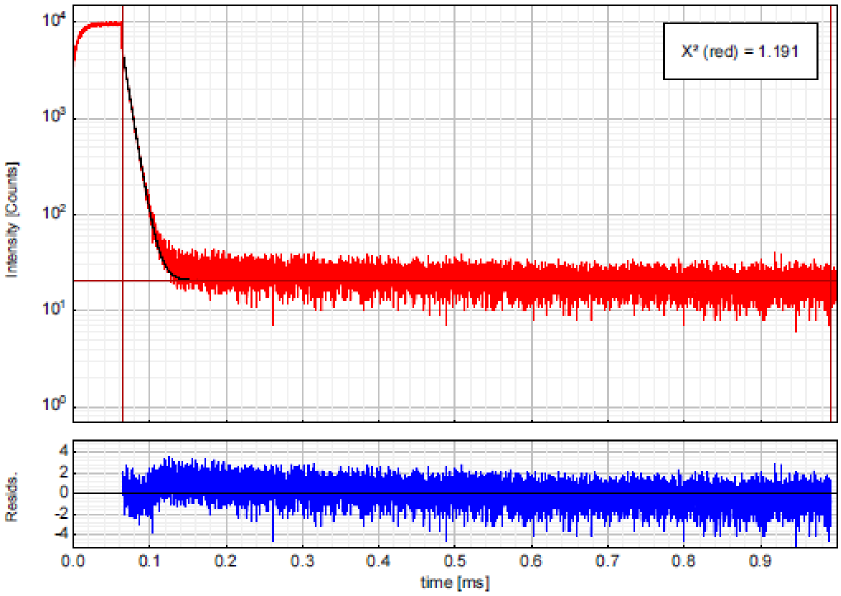

$$I(t) = \sum_{i=1}^{n} A_i \, e^{-\frac{t}{\tau_i}}$$

Parameter	Value	Conf. Lower	Conf. Upper	Conf. Estimation
A_1 [Cnts]	4457	-119	+119	Fitting
τ_1 [ms]	0.008893	-0.000182	+0.000182	Fitting
Bkgr. Dec [Cnts]	20.672	-0.843	+0.843	Fitting

Average Lifetime:

$\tau_{Av.1}$=0.008893 ms (intensity weighted)

$\tau_{Av.2}$=0.008893 ms (amplitude weighted)

Figure 4: Sm(DPA) pH = 2 luminescence lifetime in 2-methyltetrahydrofuran glass at 77 K. Fitted using the PicoQuant FluoFit software.

3.3 Sm(DPA) pH = 5

3.3.1 Sm(DPA) pH = 5 Excitation Spectrum

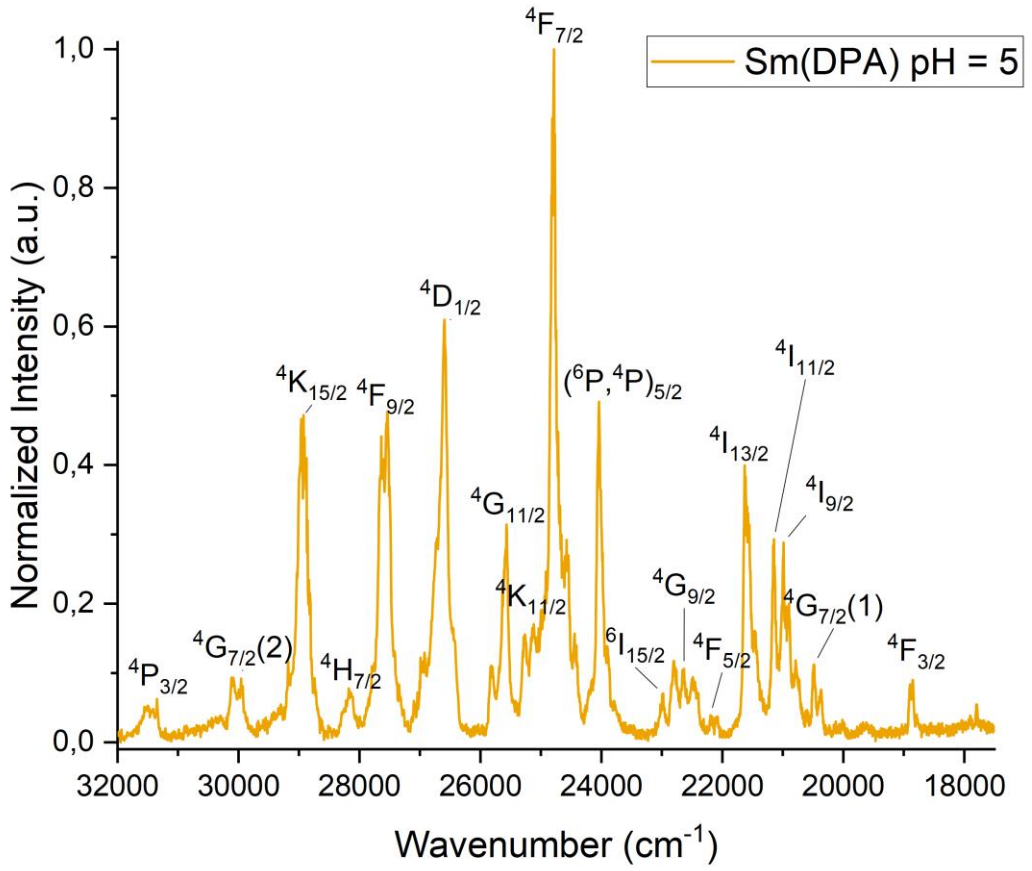

Figure 5: Normalised excitation spectra (emission at 614 nm) for Sm(DPA) pH = 5 in 2-methyltetrahydrofuran glass at 77 K.

3.3.2 Sm(DPA) pH = 5 Luminescence Lifetimes

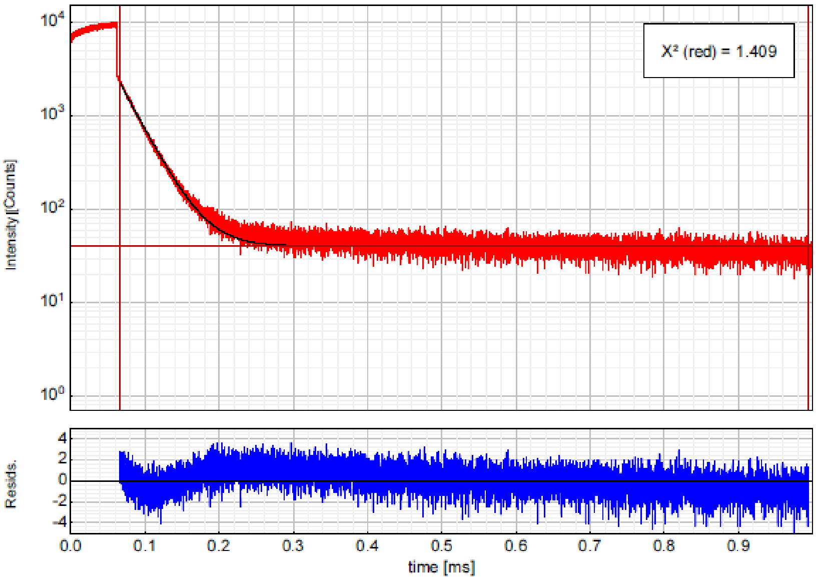

$$I(t) = \sum_{i=1}^{n} A_i e^{-\frac{t}{\tau_i}}$$

Parameter	Value	Conf. Lower	Conf. Upper	Conf. Estimation
A_1 [Cnts]	2227.1	-52.4	+52.4	Fitting
τ_1 [ms]	0.028589	-0.000553	+0.000553	Fitting
Bkgr. Dec [Cnts]	40.27	-1.30	+1.30	Fitting

Average Lifetime:

$\tau_{Av.1}$=0.028589 ms (intensity weighted)

$\tau_{Av.2}$=0.028589 ms (amplitude weighted)

Figure 6: Sm(DPA) pH = 5 luminescence lifetime in 2-methyltetrahydrofuran glass at 77 K. Fitted using the PicoQuant FluoFit software.

3.4 Sm(DPA) pH = 7

3.4.1 Sm(DPA) pH = 7 Excitation Spectrum

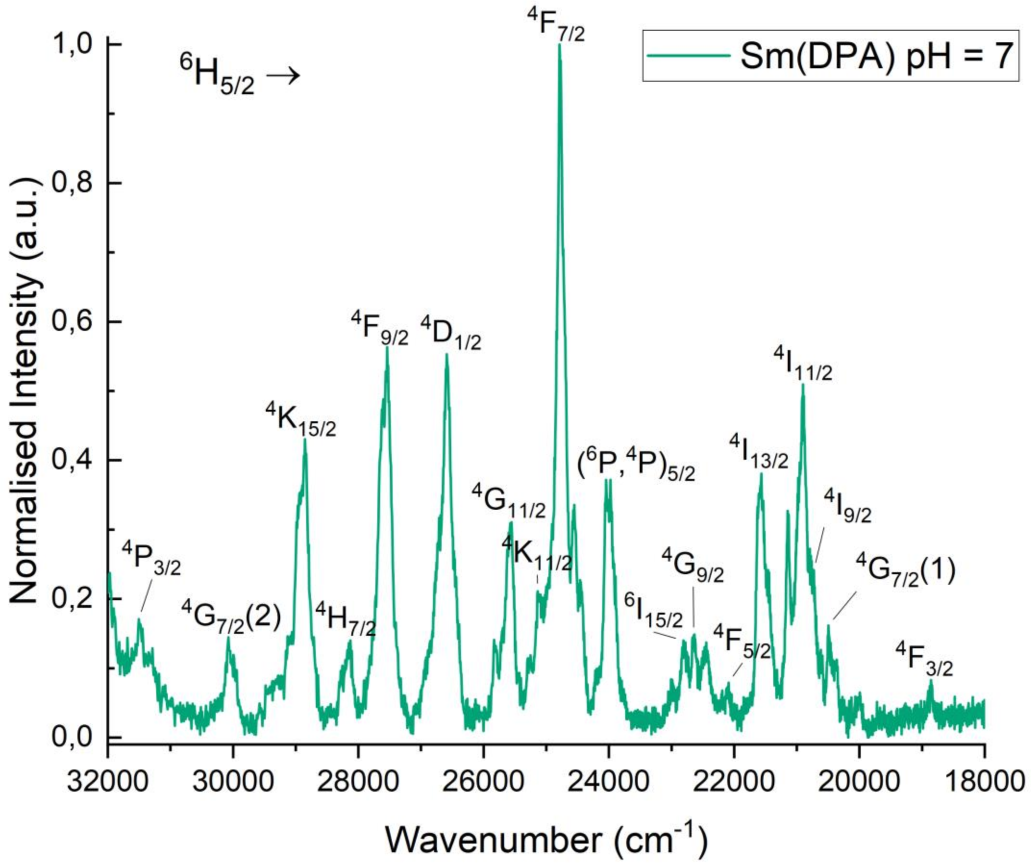

Figure 7: Normalised excitation spectra (emission at 614 nm) for Sm(DPA) pH = 7 in 2-methyltetrahydrofuran glass at 77 K.

3.4.2 Sm(DPA) pH = 7 Luminescence Lifetimes

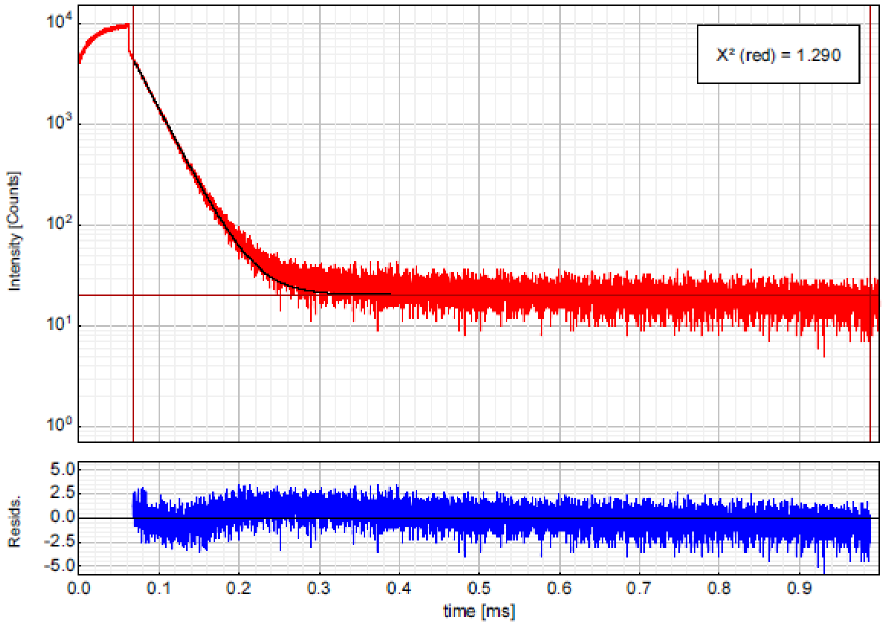

$$I(t) = \sum_{i=1}^{n} A_i e^{-\frac{t}{\tau_i}}$$

Parameter	Value	Conf. Lower	Conf. Upper	Conf. Estimation
A_1 [Cnts]	4470.9	-69.2	+69.2	Fitting
τ_1 [ms]	0.028417	-0.000338	+0.000338	Fitting
Bkgr. Dec [Cnts]	20.398	-0.927	+0.927	Fitting

Average Lifetime:

$\tau_{Av.1}$=0.028417 ms (intensity weighted)

$\tau_{Av.2}$=0.028417 ms (amplitude weighted)

Figure 8: Sm(DPA) pH = 7 luminescence lifetime in 2-methyltetrahydrofuran glass at 77 K. Fitted using the PicoQuant FluoFit software.

3.5 Sm(DPA) pH = 10

3.5.1 Sm(DPA) pH = 10 Excitation Spectrum

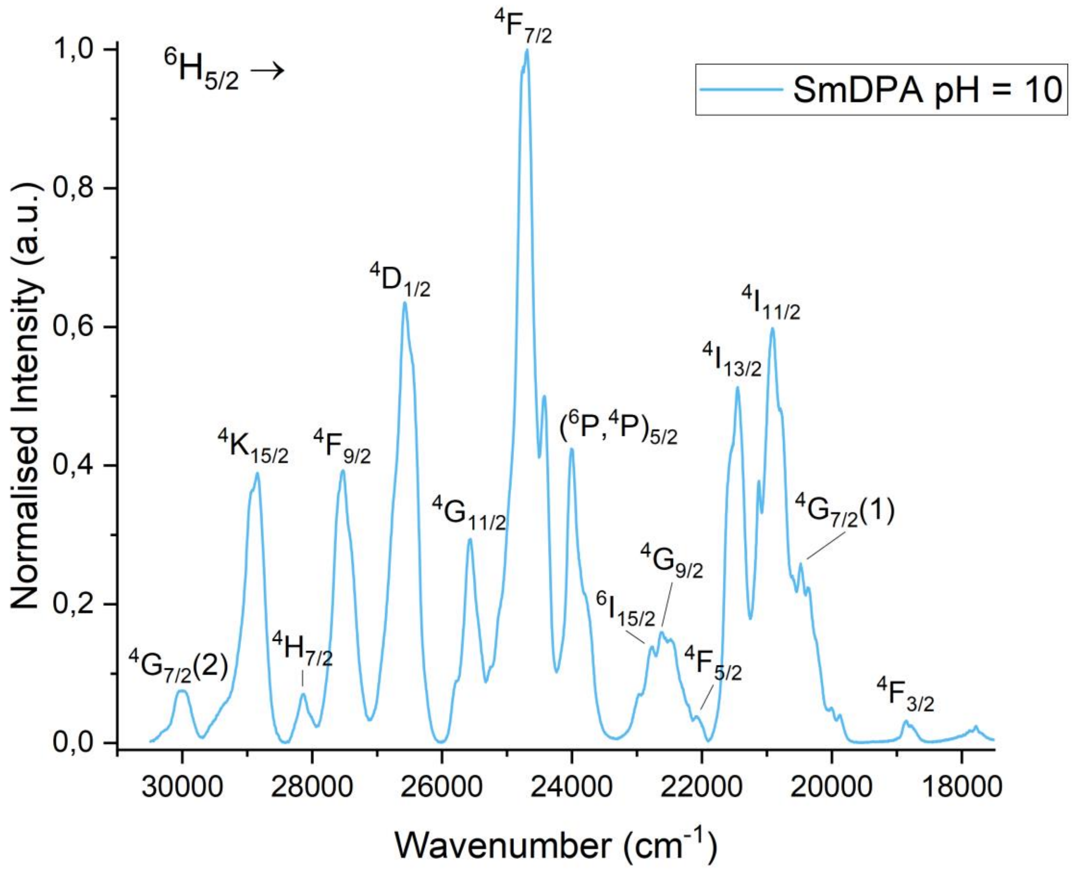

Figure 9: Normalised excitation spectra (emission at 614 nm) for Sm(DPA) pH = 10 in 2-methyltetrahydrofuran glass at 77 K.

3.5.2 Sm(DPA) pH = 10 Luminescence Lifetimes

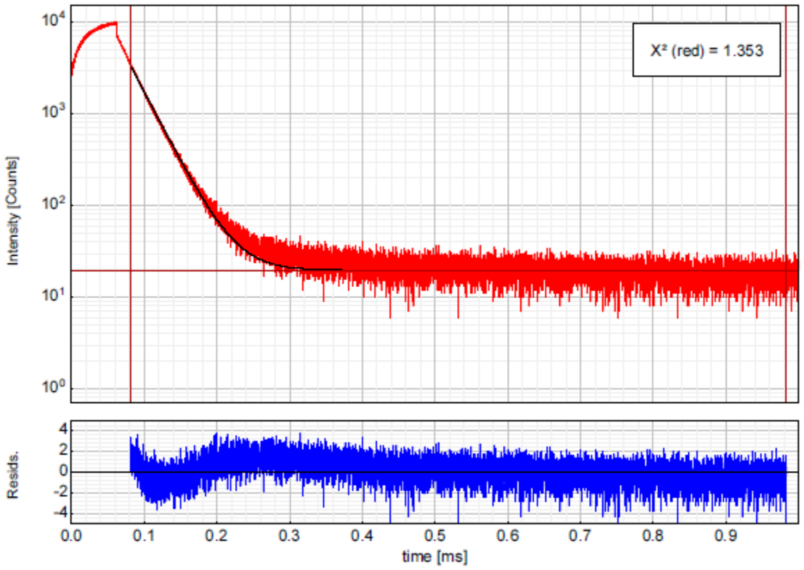

$$I(t) = \sum_{i=1}^{n} A_i e^{-\frac{t}{\tau_i}}$$

Parameter	Value	Conf. $_{Lower}$	Conf. $_{Upper}$	Conf. $_{Estimation}$
A_1 [Cnts]	3336.3	-60.9	+60.9	Fitting
τ_1 [ms]	0.028298	-0.000399	+0.000399	Fitting
Bkgr. $_{Dec}$ [Cnts]	19.661	-0.930	+0.930	Fitting

Average Lifetime:

$\tau_{Av.1}$=0.028298 ms (intensity weighted)

$\tau_{Av.2}$=0.028298 ms (amplitude weighted)

Figure 10: Sm(DPA) pH = 10 luminescence lifetime in 2-methyltetrahydrofuran glass at 77 K. Fitted using the PicoQuant FluoFit software.

4 References

Drew, M. G. (1977). *Coordination Chemistry Reviews* **24**, 179-275.

Thomsen, M. S., Anker, A. S., Kacenauskaite, L. & Sørensen, T. J. (2022).

Thomson, J. J. (1904). *The London, Edinburgh, and Dublin Philosophical Magazine and Journal of Science* **7**, 237-265.
